# Anti-Infective and Antiviral Activity of Valinomycin and Its Analogues from a Sea Cucumber-Associated Bacterium, *Streptomyces* sp. SV 21

**DOI:** 10.3390/md19020081

**Published:** 2021-02-02

**Authors:** Joko T. Wibowo, Matthias Y. Kellermann, Matthias Köck, Masteria Y. Putra, Tutik Murniasih, Kathrin I. Mohr, Joachim Wink, Dimas F. Praditya, Eike Steinmann, Peter J. Schupp

**Affiliations:** 1 Institute for Chemistry and Biology of the Marine Environment (ICBM), Carl-von-Ossietzky University Oldenburg, Schleusenstraße 1, D-26382 Wilhelmshaven, Germany; matthias.kellermann@uni-oldenburg.de; 2 Research Center for Biotechnology, Indonesian Institute of Science, Jl. Raya Bogor KM 46, Cibinong 16911, Indonesia; mast001@lipi.go.id (M.Y.P.); tuti007@lipi.go.id (T.M.); Dimas.Praditya@ruhr-uni-bochum.de (D.F.P.); 3 Alfred-Wegener-Institut für Polar- und Meeresforschung in der Helmholtz-Gemeinschaft, Am Handelshafen 12, 27570 Bremerhaven, Germany; Matthias.Koeck@awi.de; 4 Helmholtz Centre for Infection Research, Inhoffenstraße 7, 38124 Braunschweig, Germany; kathrinmohr4@gmail.com (K.I.M.); joachim.wink@helmholtz-hzi.de (J.W.); 5 TWINCORE-Centre for Experimental and Clinical Infection Research (Institute of Experimental Virology) Hannover, Feodor-Lynen-Str. 7-9, 30625 Hannover, Germany; Eike.Steinmann@ruhr-uni-bochum.de; 6 Department of Molecular and Medical Virology, Ruhr-University Bochum, 44801 Bochum, Germany; 7 Helmholtz Institute for Functional Marine Biodiversity at the University of Oldenburg (HIFMB), Ammerländer Heerstrasse 231, D-26129 Oldenburg, Germany

**Keywords:** cyclodepsipeptides, marine Actinobacteria, *Streptomyces* spp., antibiotic, sea cucumber, HCV

## Abstract

The manuscript investigated the isolation, characterization and anti-infective potential of valinomycin (**3**), streptodepsipeptide P11A (**2**), streptodepsipeptide P11B (**1**), and one novel valinomycin analogue, streptodepsipeptide SV21 (**4**), which were all produced by the Gram-positive strain *Streptomyces* *cavourensis* SV 21. Although the exact molecular weight and major molecular fragments were recently reported for compound **4**, its structure elucidation was not based on compound isolation and spectroscopic techniques. We successfully isolated and elucidated the structure based on the MS^2^ fragmentation pathways as well as ^1^H and ^13^C NMR spectra and found that the previously reported structure of compound **4** differs from our analysis. Our findings showed the importance of isolation and structure elucidation of bacterial compounds in the era of fast omics technologies. The here performed anti-infective assays showed moderate to potent activity against fungi, multi drug resistant (MDR) bacteria and infectivity of the Hepatitis C Virus (HCV). While compounds **2**, **3** and **4** revealed potent antiviral activity, the observed minor cytotoxicity needs further investigation. Furthermore, the here performed anti-infective assays disclosed that the symmetry of the valinomycin molecule is most important for its bioactivity, a fact that has not been reported so far.

## 1. Introduction

Natural products play a dominant role in the discovery of leads for the development of novel drugs to treat human diseases [1]. For the past 30 years, there has been an increasing effort by scientists from many disciplines to identify novel natural products from marine organisms, including marine bacteria, due to their rich biological and chemical diversity [2,3]. With the strong demand to find new antibiotics to solve the antibiotic resistance crisis, research on marine natural products expanded in the last decade to include marine bacteria. Several studies have shown that many novel bioactive compounds were often derived from marine Gram-positive Actinobacteria [3,4]. 

Recently, the isolation and fast identification of putatively new bioactive compounds from sea cucumber-associated bacteria have been reported in [5]. Extracts of *Streptomyces* sp. SV 21 showed potent anti-infective activities. Bio-guided fractionation of the bacterial extracts and subsequent MS/MS and NMR experiments revealed valinomycin (**3**), two of its analogues, namely streptodepsipeptide P11B (**1**), streptodepsipeptide P11A (**2**) [6], and one putative new valinomycin analogue named streptodepsipeptide SV21 (**4**). All the compounds (**1**–**4**) exhibited inhibitory activities against bacteria, fungi, and the Hepatitis C Virus (HCV). Streptodepsipeptide SV21 (**4**) had recently been characterized based on exact mass and MS/MS analysis [7]; however, it was neither isolated nor elucidated via NMR nor tested for its bioactivities.

Valinomycin is a common cyclodepsipeptide produced by various soil-derived Actinobacteria, such as *Streptomyces fulvissimus, S. roseochromogenes,* and *S. griseus* var. *flexipartum* [8], as well as from marine *Streptomyces* species associated with the sponges *Axinella polypoides* and *Aplysina aerophoba* [9]. Valinomycin is considered as one of the ionophore antibiotics. This bioactive compound has both a hydrophobic and hydrophilic moiety, which is necessary to bind and shield ions, but also allows the molecule to transport those ions through the lipophilic membrane barrier of living cells. Valinomycin is known to be highly selective for binding potassium ions and thus has the potential to disrupt the intracellular ion concentration of the cell. The probability to transport ions through membranes not only affects osmoregulatory processes, but also affects the homeostasis of the cell, which in turn may result in an increased level of toxicity or even death for the organism [10,11]. Therefore, this cyclodepsipeptide was reported to have many bioactivities, such as antitumor, antibacterial, antibabesia, and antifungal activity [6,9,12,13]. Another interesting bioactivity of valinomycin was its potency against the causative agent of the world’s first pandemic in the 21st century, the SARS-CoV virus. Unfortunately, valinomycin also showed enhanced cytotoxicity that prevented the drug to enter the clinical phase [14]. Besides the pharmacological potential, an ecological role also has been reported, as valinomycin is used in chemical defense against pathogens by the leafcutter ant *Acromyrmex echinatior* [15]. 

In this study, valinomycin (**3**) and its three analogues (**1**, **2**, and **4**) were isolated and characterized based on MS/MS and NMR analysis. In addition, the anti-infective activities of compounds **1**–**4** against multi drug-resistant (MDR) bacteria (*Bacillus subtilis*, *Staphylococcus aureus*), fungi (*Candida albicans*, *Mucor hiemalis*, *Rhodoturula glutinis*) and the Hepatitis C Virus (HCV) were identified. 

## 2. Results and Discussion

The exact masses of the compounds (MS^1^ data, Figure S1) were compared with compounds in the MarinLit database (Table 1). The results showed that exact masses of compounds **1**–**3** closely matched with streptodepsipeptide P11B ([M] = 1082.5988), P11A ([M] = 1096.6142), and valinomycin ([M] = 1110.6315), respectively. While, streptodepsipeptide SV21 (**4**), having a precursor ion of *m/z* 1142.6804 [M + NH_4_]^+^, had not been reported yet in the MarinLit database. Further analysis, using the GNPS MASST database, on the precursor (MS^1^) and product ion (MS^2^) spectral data (Figures S2–S5) showed that compounds **1**–**4** were related to valinomycin with cosine scores ranking all above 0.7, while considering more than 40 major product ion peaks. This result indicated that **4** was also a valinomycin analogue. 

To have an overview of the structure for all four valinomycin analogues, we analyzed and compared the MS^2^ spectra of compounds **1**, **2**, and **4** with valinomycin (**3**). Valinomycin consists of the enantiomers *D*- and *L*-valine (Val), *D*-α-hydroxyisovaleric acid (Hiv), and *L*-lactic acid (Lac) [6]. One valinomycin molecule consists out of three repeating units of Val–Hiv–Val–Lac, where one unit has an exact mass of 370.208 Da. 

Compound identification started by comparing the precursor ion of each compound (cf. Table 1) as well as the fragment ions in the mass region between *m/z* 600 and *m/z* 1150 (Figure S6). Both, the precursor ion and the fragment ions showed a consecutive mass increase of 14 Da from compound **1** to **4**. However, the fragment ion *m/z* 713.4 was found to be in all valinomycin analogues. Based on the molecular mass, the fragment ion at *m/z* 713.4 is represented by two units of Val–Hiv–Val–Lac after the loss of a unit of C=O for the initial opening of the ring structure. To crosscheck the existence of a unit Val–Hiv–Val–Lac in all compounds, the neutral loss of 370.208 Da was observed in all compound spectra after the loss of a C=O unit, which initially opened the ring structure. The neutral loss of two units of Val–Hiv–Val–Lac, with an exact mass of *m*/*z* 740.419 Da, was also observed in compounds **1**–**4**, indicating that all four molecular species contained at least two units of Val–Hiv–Val–Lac.

Despite the similar smaller fragment ions that occurred in the mass spectra at *m/z* 50–*m/z* 600, further analysis showed some unique fragment ions for each valinomycin analogue. For example, the fragment ion at *m/z* 315.192 was only present in streptodepsipeptide P11B (**1**). This compound represents a monomer and dimer of depsipeptide (Val–Hiv–Val–Lac) as well as a monomer of Val–Hiv–Val–Lac minus 28 Da. Based on the previous studies, the mass difference of 28 Da might be the result of the substitution of a Hiv with a Lac [6,7]. Therefore, the fragment ion at *m/z* 315.192 is likely a unit of Val–Lac–Val–Lac. This is further supported by the neutral loss of 171 Da (Val–Lac) from *m/z* 315.192 to *m/z* 144.103 (shown as black arrow in Figure S6A). The fragment ion at *m/z* 144.103 is a unit of Val–Lac with a loss of C=O. 

Another example is represented by the fragment ion at *m/z* 329.208, which was only found in streptodepsipeptide P11A (**2**). This compound has a mass of about 14 Da lower than a unit of Val–Hiv–Val–Lac. By comparing out the MS^2^-based analysis with previous studies, there are at least two possible explanations: first, a substitution of a Hiv with a hydroxybutanoic acid (Hba) [6]; or second, a substitution of a Val with an isoleucine or a leucine (Ile/Leu) in a unit of Val–Lac–Val–Lac [7]. Both scenarios would be possible since the ion for Val–Hba–Val–Lac and Ile/Leu–Lac–Val–Lac create a fragment mass of *m/z* 329.207. Furthermore, the fragment ion at *m/z* 186.113 in **2** might be the protonated ion of Val–Hba or Ile/Leu–Lac. However, after the fragmentation pathway of the compound was simulated for both substitutions, a peak for Val–Lac–Val–Lac around *m/z* 315.191 could not be found in the spectra, if a Val was substituted with Ile/Leu. Therefore, the most suitable substitution in **2** is a Hiv with a Hba, as also mentioned in [6]. 

**Figure 1:**
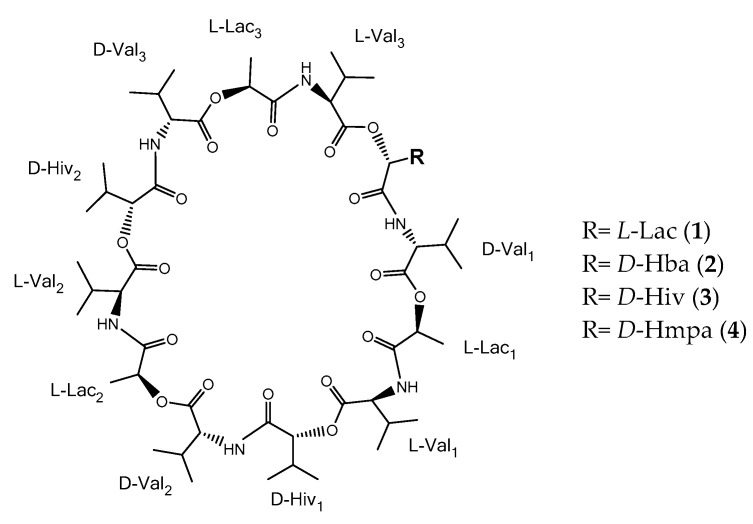
Identified structures of the four valinomycin analogues **1**–**4**. Based on our NMR and MS^2^ experiments, we assumed the absolute configuration to be identical to the one reported by Ye and colleagues [6].

A fragment ion at *m/z* 357.239 occurred in the spectra for streptodepsipeptide SV21 (**4**). It has a molecular mass of 14 Da higher than a unit Val–Hiv–Val–Lac. In a previous study, the additional 14 Da were suggested to result from a substitution of a Val with either an Ile or Leu unit [7]. However, when we simulated the fragmentation of Val–Hiv–Ile/Leu–Lac, we were unable to detect the peak at *m/z* 158.118 in the MS^2^ spectra. Fragmentation of Val–Hiv–Ile/Leu–Lac should have resulted in Val–Hiv and Ile/Leu–Lac with the calculated *m/z* 172.133 and *m/z* 158.118, respectively. Therefore, we propose the structure of **4** to have a substitution of Hiv with hydroxymethylpentanoic acid (Hmpa), since we detected the fragment ions *m/z* 186.150 (Val–Hmpa) and *m/z* 144.103 (Val–Lac), thus indicating a single unit of Val–Hmpa–Val–Lac. In summary, the difference between compounds (**1**–**4**) is only a substitution of a Hiv with either a Lac, a Hba or Hmpa within a single unit of Val–Hiv–Val–Lac (cf. Figure 1). 

The configuration of the compounds **1**–**3** in Figure 1 was assumed to be identical with [6], while configuration of **4** was derived from the biosynthetic pathway of valinomycin. The structure of compounds **1**–**4** were quite similar, therefore we agreed with [7] that valinomycin and their analogues are derived from the same biosynthetic pathway. Biosynthesis of valinomycin is accomplished by nonribosomal peptides (NRPS) that are composed of two proteins, namely, VLM1 and VLM2. Those proteins are divided into four modules, each one responsible for incorporation of one unit of *D*-Hiv, *D*-Val, *L*-Lac, and *L*-Val. The depsipeptide chain (*D*-Hiv–*D* -Val–*L*-Lac–*L*-Val) is linked to the C-terminal iterative thioesterase (TE) domain at the last module in VLM2. The terminal TE domain controls the termination, release and cyclization of the growing chains in the biosynthetic process [16].

Biosynthesis of *D*-Hiv in valinomycin is occurring in module 1 in VLM1 [16]. The study also explained that module 1 contains four functional domains: adenylation (A; designated as VLM1A1), hypothetical transaminase (TA), hypothetical dehydrogenase (DH2) and peptidyl carrier protein (PCP). Extracted NRPS codes from VLM1A1 did not yield any predictable substrate, leading to the assumption that VLM1A1 might have adapted to select and activate hydroxyl acids independently [16]. However, the adenylation domain is a core of each module that recognize the cognate substrate [17]. Therefore, feeding experiments using different substrates, i.e., *D*-Hiv, *D-*Hba, or *D-*Hmpa, are needed to verify which substrates are needed to produce valinomycin and its analogues. In turn, substitution of *D-*Hiv with *L-*Lac in streptodepsipeptide P11B (**1**) could be explained as a variation in the linearity within modules 3 and 4 instead of modules 1 and 2 in one round of the tetradepsipetide assembly [7].

**Figure 2:**
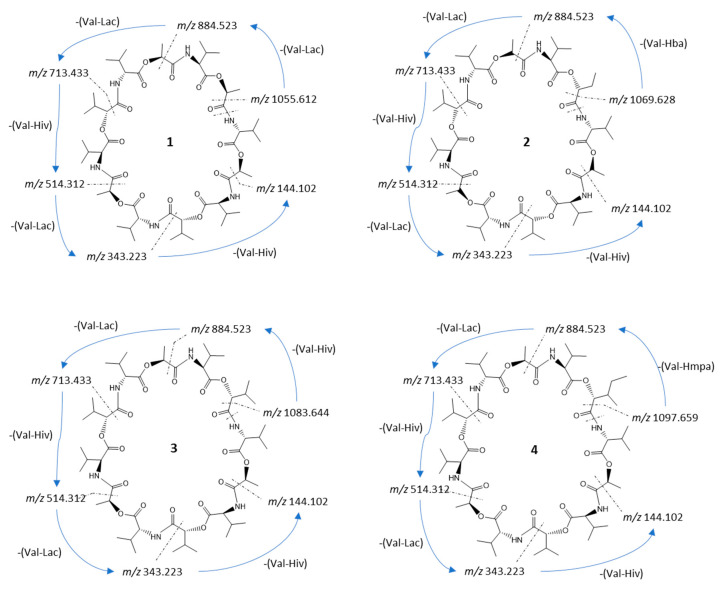
Scheme of the fragmentation pathways of compounds **1**–**4**. The *m/z* ratios were calculated and then compared with the MS^2^ fragment ions. The fragmentation started from the loss of a C=O unit.

Streptodepsipeptide P11B (**1**) proved to be the known depsipeptide based on the comparison of the measured MS, NMR, and optical rotation data with the references [6,7]. To crosscheck the structure with the MS^2^ data, we simulated the fragmentation pathways for each compound. The fragmentation pathway of compound **1** started with the ring opening and the loss of a C=O unit of 27.995 Da. After the ring opened, the fragment ion *m/z* 1055.611 (calc. *m/z* 1055.612) (Figure 2) continued to lose either a unit of Val–Lac or Val–Hiv with 171.089 and 199.121 Da, respectively (cf. Figure S7). The sequential loss of a unit of Val–Lac or Val–Hiv explained the occurrence of fragment ions *m/z* 884.520 (calc. *m/z* 884.523) or *m/z* 856.491 (calc. *m/z* 856.491; fragment ions from MS^2^ spectra *cf.* Figure S7). 

In total, the fragmentation pathway of streptodepsipeptide P11B (**1)** took five major steps of losing either a unit of Val–Lac or Val–Hiv. However, since **1** has one substitution of Hiv with a Lac residue, one of the fragmentation steps is the repetition of losing a unit of Val–Lac, which is indicated by the consecutive tan arrows in Figure S7.

**Figure 3:**
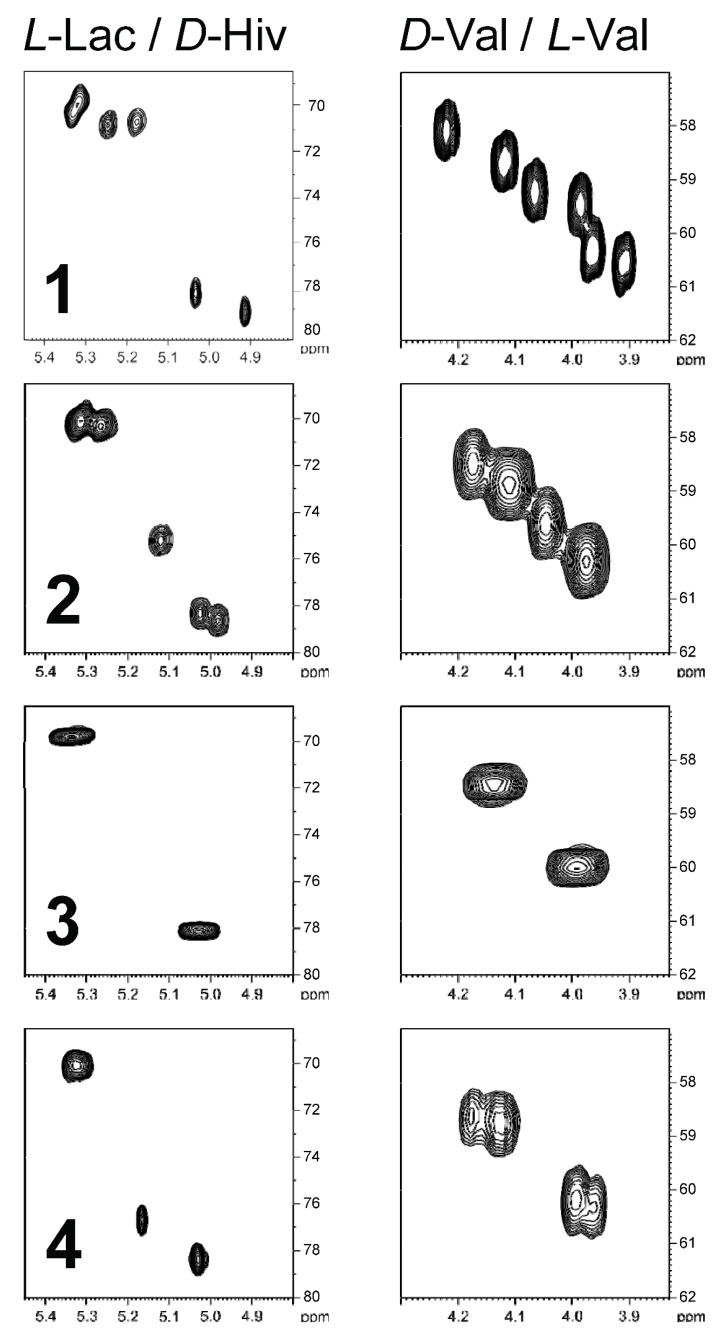
The Hα/Cα region of the HSQC spectra of compounds **1**–**4** for *D*-Hiv/*L*-Lac on the left side and *D*-Val/*L*-Val on the right side. The spectra on the top represent the ones for streptodepsipeptide P11B (**1**) and the ones on the bottom for **4**, respectively. The *D*-Hiv/*L*-Lac plots (left side) are divided into two subgroups: appr. 71 ppm (*L*-Lac) and appr. 79 ppm (*D*-Hiv). The same is true for the *D*-Val/*L*-Val plot (right side): appr. 59 ppm (*D*-Val) and appr. 60 ppm (*L*-Val).

Besides a thorough mass spectral analysis, the four valinomycin analogues were also compared by their Hα/Cα region of the HSQC spectra (Figure 3). Every residue showed a distinct fingerprint region in the HSQC spectrum and thus allows counting the different residues. For example, in the spectra of streptodepsipeptide P11B (**1**), all 12 Hα/Cα correlations can be observed. The *D*-Val/*L*-Val plot indicates no changes since there are three signals for *L*-Val and three signals for *D*-Val. However, the plot on the left side indicates a loss of a Hiv residue and an increase of a Lac residue, which is in accordance with the molecular formula of **1** (valinomycin minus 2 × CH_2_). In case of streptodepsipeptide SV21 (**4**), there is quite some overlap in the four regions of the HSQC spectrum. Two of the three *L*-Val and two of the three *D*-Val residues overlapped. The same is true for the two Hiv residues. The three Lac residues all appear in one signal. The only signal without any overlap is the new Hmpa residue (5.17 ppm/76.7 ppm).

The precursor ion of streptodepsipeptide P11A (**2**) was 1114.649 [M + NH_4_]^+^. It has 14 Da more compared to streptodepsipeptide P11B (**1**). Most of the fragment ions of **2** also have a 14 Da difference to fragment ions of **1** (cf. Figure S6). Therefore, the fragment ion at *m/z* 1069.627 (calc. 1069.628) resulted from a neutral loss of a C=O (Figure 2). The loss of a C=O may have happened anywhere in the structure; therefore, fragmentation of **2** then continued with a loss of a unit of Val–Lac, Val–Hiv, or Val–Hba at any positions near the opened ring. It explains the occurrence of the fragment ion *m/z* 898.536 (calc. 898.538), *m/z* 884.524 (calc. 884.523), *m/z* 870.509 (calc. 870.507), etc. (cf. Figure S8). Fragmentation pathways for **2** in Figure S8 seems to be more complex than **1**. However, the pathways still consist of five major steps of losing intermittently a unit of Val–Lac then Val–Hiv, or Val–Lac then Val–Hba, with only the loss of one Val–Hba for each possible pathway (blue arrows in Figure S8). 

The reported fragmentation pattern for streptodepsipeptide P11A (**2**) in [7] did not fit well with our measured fragment ions of compound **2**. However, the NMR data for streptodepsipeptide P11A (**2**) (Figure 3) matched closely with the published data in [6] and also resembled our fragmentation pattern (cf. Figure S8).

The exact mass and also precursor ion (±0.01 Da) of the isolated valinomycin (**3**) matched the reported mass data in [6,7,9]. The fragmentation of **3** is initiated by the loss of a C=O at any position in the ring to produce the fragment ion *m/z* 1083.643 (calc. 1083.644). The fragmentation process is then followed by the loss of a unit of Val–Lac or Val–Hiv. Following the same five major fragmentation steps as for streptodepsipeptide P11B (**1**) and P11A (**2**), fragment ions *m/z* 912.549 (calc. 912.554), *m/z* 884.524 (calc. 884.523), *m/z* 713.433 (calc. 713.433), etc., were detected (cf. Figure S9). 

Streptodepsipeptide SV21 (**4**) had a precursor ion of *m/z* 1142.680 [M + NH_4_]^+^ and did not match any reported data in MarinLit. However, MS^2^ analysis of **4** using the MASST GNPS database showed that **4** was strongly related to valinomycin based on a cosine score of 0.77 and 42 shared peaks (Figure S5). A cosine score has a value between 0 and 1, with 1 indicating 100% similarity. A sample is considered an analogue of a reported compound if the cosine score is >0.7 [18]. Several measured peaks of **4** were identical to the fragment ions of valinomycin (**3**) (visualized by green lines, see Figures S2–S5). Fragment ion *m/z* 1097.660 (calc. *m/z* 1097.659) resulted from the loss of a C=O group via ring opening. The fragmentation process then followed the previous reported pattern via loss of Val–Lac, Val–Hiv, or Val–Hmpa, resulting in peaks of *m/z* 926.566 (calc. *m/z* 926.570), *m/z* 898.540 (calc. *m/z* 898.538), and *m/z* 884.523 (calc. *m/z* 884.523), respectively (cf. Figure S10). 

For the ^13^C and ^1^H NMR assignments of valinomycin (**3**), please see Figure 3. The structure of valinomycin consisted of four units: *L*-Val (appr. 60 ppm), *D*-Val (appr. 59 ppm), *L*-Lac (appr. 71 ppm), and *D*-Hiv (appr. 79 ppm). Each unit showed characteristic NMR signals, which were useful for the characterization of the analogues (**1**, **2**, and **4**). The ^1^H NMR spectra showed the purity of compounds **1**–**4** and allowed the comparison with the reported compounds from [6] (cf. Figures S11–S15 and Tables S1–S4). 

The ^13^C NMR signals for streptodepsipeptide SV21 (**4**) displayed 55 carbon signals for 12 carbonyls (δ_C_ 169.9–172.4), six oxymethines (δ_C_ 19.1–19.3), six nitrogenated methines (δ_C_ 118.0–118.8), nine methines (δ_C_ 19.0–26.1), a methylene (δ_C_ 14.0), and 21 methyls (Table 2 and Figure S16). The ^1^H NMR spectrum of **4** showed six signals for NH at δ_H_ 7.88, 7.83, 7.80, 7.75, 7.69, and 7.67 ppm (Table 2 and Figure S17). Those data indicated that **4** consisted of 6 esters and 6 amino acids residues. 

The MS results clearly indicate that compound **4** has an additional methylene group compared to valinomycin (**3**). In principle, the additional CH_2_ group could be added to each of the four residues. The inspection of the four Hα/Cα regions in the HSQC spectrum (cf. Figure 3) clearly shows a loss of a Hiv residue, which means that the extra CH_2_ group of **4** was added to this residue. There are two possibilities how to add a methylene group: First, a transition from Hiv to Hmpa would be possible. Second, from an “amino acid” point of view, it could be the transition from Val to Leu or Ile. However, the Hmpa unit was established by analysis of the COSY and HMBC spectra. The complete spin system of Hmpa could be assigned by the TOCSY spectrum without the determination of the explicit positions. Starting from Hα, the Hβ can be assigned by the COSY correlation, and the corresponding Cβ (36.7 ppm) is accessible by the HSQC (Figure 3 and Figure S18). Furthermore, three HMBC correlations can be observed starting from Hα (36.7 ppm, 26.1 ppm, and 14.0 ppm). This already indicates the existence of a Hmpa residue. For the constitutional isomer “Leu” only two HMBC correlations would have been expected and no correlation to a methyl group (here 14.0 ppm). The complete assignment of the Hmpa residue is given in Table 2.

Valinomycin (**3**) and its analogues (**1**, **2**, and **4**) showed a narrow spectrum of antimicrobial activities (Table 3). Compounds **1**–**4** showed that all antifungal activity against *Mucor hiemalis (Mh)* and *Ruegeria glutinis (Rg)*, with streptodepsipeptide P11A (**2**) and valinomycin (**3**) being the most active ones, revealed similar or lower MIC values compared to the commercial antifungal compound nystatin (cf. Table 3). Valinomycin was eight times stronger than nystatin against *Mh* and as strong as nystatin against *Rg.* Previous studies had also shown antifungal activity of valinomycin against the plant pathogens *Phytophthora capsici* and *Botrytis cinerea* [13,14]. Only valinomycin (**3**) and streptodepsipeptide SV21 (**4**) also exhibited activities against the Gram-positive bacterium *Staphylococcus aureus (Sa)* and *Bacillus subtilis* (*Bs*) (only (**4**) had activity). However, the activity of valinomycin on *Bs* was strongly affected by the pH regime in the conducted assay. At the different pH values from 5.5 to 9.5, valinomycin showed an increase in antibacterial activity against *Bs* at higher pH values [19]. To the best of our knowledge, this is the first report on the antimicrobial activity of compounds **1**, **2**, and **4**.

**Figure 4:**
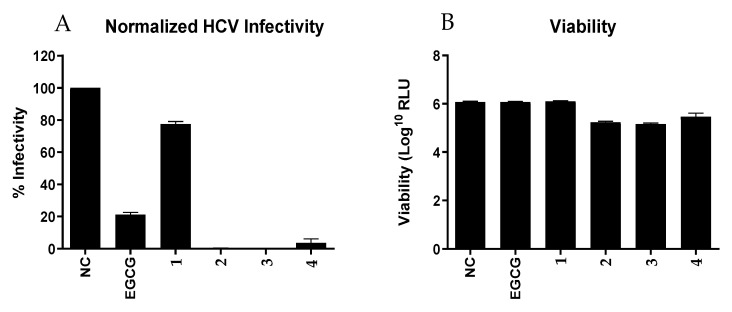
Activity of compounds **1**–**4** to Huh7.5 cells that were infected with Hepatitis C Virus (HCV). (**A**) The percentage of HCV-infected hepatoma cells. (**B**) The viability of Huh7.5 cells after exposure to the test compounds. NC: negative control; and EGCG: epigallocatechin gallate as positive control.

Furthermore, we tested all 4 compounds (**1**–**4**) against the Hepatitis C Virus (HCV, Figure 4). Although structurally closely related, only the compounds **2**–**4** showed pronounced infectivity against HCV compared to the positive control epigallocatechin gallate (EGCG). Streptodepsipeptide P11B (**1**) significantly less affected the HCV. This finding indicated that valinomycin (**3**) and its analogues (**2** and **4**) have a strong potential to function as potent ani-HCV agents. However, viability of the Huh7.5 cells for valinomycin (**3**) and its analogues (**2** and **4**) were lower than for the positive control, meaning these compounds also slightly affected the host cell as well.

The isolation and structure assignment of valinomycin and the three derivatives, including the new streptodepsipeptide SV21 (**4**), allowed us for the first time to conduct a structure activity relationship (SAR) analysis to determine the essential functional groups for the observed antimicrobial and newly reported antiviral activities. The symmetry of the ring system seems to be key for the activity of valinomycin and its analogues. Compounds **1**–**4** have the same number of carbonyl groups and share the same two units of Val–Hiv–Val–Lac. The difference is only in one unit of the depsipeptide, which affects the symmetry of the molecule. Valinomycin affects the cells by dissipating the electrochemical gradient, which is essential for cell life through influx of the potassium ions into the cell. Valinomycin can change its conformation, allowing it to dissolve in aqueous but also lipophilic environments. The carbonyl groups in valinomycin form the hydrophilic site, while the methyl and isopropanyl groups form the hydrophobic site. A previous study on the antibiotic mechanism of valinomycin indicated that potassium was released from the hydrophilic site after forming a complex with valinomycin. At the hydrophobic membrane interface, the potassium ion is selectively released through the substitution with water molecules at the Lac rather than Hiv site [20].

Valinomycin (**3**) was the most active of the four tested compounds. Deletion of one or two CH_2_ groups in the valinomycin structure resulted in weaker antifungal activity, while addition of a CH_2_ group gave weaker antifungal but broader antibacterial activity. Thus, it seems likely that the symmetry of valinomycin is important for the observed higher bioactivity. 

The antiviral activity of valinomycin (**3**) and its analogues (**2** and **4**) against HCV amplifies the potential of these compounds to be developed as or used a scaffold for the development of anti-viral agents. In a previous study, valinomycin also showed promising activity against the SARS-CoV virus [21]. Therefore, it will be interesting to conduct experiments with valinomycin and its analogues against the new strain of Coronavirus (SARS-CoV-2).

## 3. Materials and Methods 

### 3.1. Bacterium Culture Condition, Isolation, and Identification of Bioactive Compounds

*Streptomyces* sp. SV 21 was isolated from the sea cucumber *Stichopus vastus* in Lampung, Indonesia. Sequences from the 16S rRNA of the bacterium has been submitted to the NCBI database under the accession number MK696479. It showed 100% similarity sequence to *Streptomyces cavourensis* with the type strain accession number NR_043851.1. Further information on bacterial isolation can be found in [5]. After successful isolation, the bacterium was grown from the glycerol stock on Marine Agar that was made from Marine Broth (MB, Carl Roth, Karlsruhe, Germany) according to manufacturer’s instruction, with addition of 9 g/L agar (Agar-agar Bacteriological, Carl Roth, Karlsruhe, Germany). A single colony of the bacterium was transferred into 10 mL of MB and incubated at room temperature (~22 °C) for three days and used as seeding broth. For that, 1 mL of the seed broth was used to inoculate 800 mL of fresh MB media stored in a sterile 2 L Erlenmeyer flask. The culture was grown for 14 days at room temperature (~22 °C) under constant shaking (100 rpm) to keep the media aerated. 

A total of 2.4 L of broth culture were filtered through a 65 g/m^2^ paper filter grade 3 hw (Sartorius, Goettingen, Germany) to separate the cell mass from the remaining MB media. Subsequently, the resulting cellular material on the paper filter was extracted exhaustively with methanol (MeOH; HPLC grade VWR International GmbH, Darmstadt, Germany). The remaining broth media was extracted with ethyl acetate (EtOAc; HPLC grade VWR International GmbH, Darmstadt, Germany) in a 1 to 1 ratio by using an Ultra-Turrax (T25, IKA, Staufen, Germany) set at 12,000 rpm and applied for approximately 30 s. Both organic extracts were finally mixed and dried using a rotary evaporator (Rotavapor R II, Büchi, Flawil, Switzerland). 

The bacterial crude extract was fractionated into seven fractions (Fr.) using a C_18_-based solid phase extraction (SPE) cartridge (10 g) with a column capacity of 75 mL (HyperSep C_18_, Fischer Scientific, Leicestershire, UK) with a mixture of DI water (Arium 611, Sartorius, Goettingen, Germany) and MeOH in different ratios starting with 70% and ending with 100% MeOH. However, only Fr. 3, 4 and 5 (85, 90, and 95% MeOH) showed high anti-infective activities. The target compounds were further purified by high-performance liquid chromatography (HPLC) (Agilent 1260 Infinity, Agilent, Santa Clara, SA, USA), using a C_18_ (Pursuit XRs 5 µm C_18_ 250 × 10.0 mm, Agilent, Santa Clara, SA, USA) semi-preparative column. A linear gradient (sequence: 10 min from 70:30 MeCN:H_2_O to 100:0 MeCN:H_2_O, 30 min 100:0 MeCN:H_2_O, then 20 min 70:30 MeCN:H_2_O) was applied. Formic acid (FA, 98%, Carl Roth, Karlsruhe, Germany) was added at a concentration of 0.1% to both the solvent MeCN and H_2_O. The flow rate was set at 1.5 mL min^−1^, the column temperature was set at 40 °C, and the detection wavelength of the Diode Array Detector (DAD; Agilent 1260 Infinity Diode Array Detector (G4212-60008), Agilent, Santa Clara, SA, USA) was set to 210 nm. With each semi-preparative run, 100 µL of a 10 mg mL^−1^ solution was injected. The target compounds eluted between 28 and 36 min. Each individual compound was collected: compound **1** (0.6 mg, RT 29.1 min), compound **2** (1.3 mg, RT 30.4 min), compound **3** (23.2 mg, 32.5 min), and compound **4** (1.8 mg, 34.6 min). 

Analysis of the SPE fractions was performed on an ultra-high-performance liquid chromatography–high-resolution mass spectrometer (UPLC-HRMS; Waters Synapt G2-Si, Milford, MA, USA). Chromatographic separation was achieved on a BEH C_18_ column (Waters ACQUITY, Milford, MA, USA; 1.7 μm, 2.1 × 50 mm) with the column temperature set to 40 °C. The mobile phase was a linear gradient (sequence: 30 min from 5:95 MeCN:H_2_O to 95:5 MeCN:H_2_O, 10 min 95:5 MeCN:H_2_O, then 10 min 5:95 MeCN:H_2_O). The flow rate was set at 0.3 mL min^−1^. Formic acid (FA, 98%, Carl Roth, Karlsruhe, Germany) was added at a concentration of 0.1% to both the solvent MeCN and H_2_O. Analytes were detected by ESI-MS in the positive ionization mode (POS) by monitoring the mass range of *m/z* 50 to 2000 Dalton (Da). The target compounds showed clear peaks in the total ion chromatogram (TIC), with *m/z* of 1100.633, 1114.650, 1128.673, and 1142.685 Da. 

For structure verification purposes, we analyzed the MS^2^ data by using MASST GNPS to identify the likely fragments of the target compounds or its analogues with the set parameters described in [5]. In addition, we also measured the NMR spectra on a Bruker 700 MHz cryo NMR spectrometer (Avance III HD) with Topspin 3.6.2 for analysis. For compounds **1** to **3**, the structures were verified with the following NMR experiments: 1D proton, ^1^H,^13^C-HSQC (pulse program: hsqcedetgpsisp2.3) and ^1^H,^13^C-HMBC (pulse program: hmbcgplpndqf). For structure elucidation of compound **4**, the following additionally experiments were conducted: 1D carbon, ^1^H,^1^H-COSY (pulse program: cosygpppqf) and ^1^H,^1^H-TOCSY (pulse program: mlevphpp). The following parameters were used for compound **4**: COSY (data acquisition: 2K/512 points, relaxation delay D1 1.5 s, acquisition time AQ 133 ms, number of scans NS 32), TOCSY (data acquisition: 2K/512 points, relaxation delay D1 2 s, acquisition time AQ 133 ms, number of scans NS 32), HSQC (data acquisition: 2K/256 points, relaxation delay D1 2 s, acquisition time AQ 146 ms, number of scans NS 16, delay ^1^*J*_CH_ 145 Hz) and HMBC (data acquisition: 2K/256 points, relaxation delay D1 1.5 s, acquisition time AQ 146 ms, number of scans NS 40, delay *^n^J*_CH_ 8 Hz). All samples were dissolved in 0.6 mL CDCl_3_. Optical rotation was measured on a polarimeter (Anton Paar, Graz, Austria) with a 100 mm path length and sodium D line at 589 nm. 

Streptodepsipeptide P11B (**1**): white amorphous powder; molecular formula C_52_H_86_N_6_O_18_; UV (MeOH, photodiode array), *λ*max 220 nm; ${\left[\alpha \right]}_{D}^{20}$ +23.3° (*c* 0.03, CHCl_3_) (lit. +26.7°, [6]); HR-ESI-MS *m/z* 1100.6332 [M + NH_4_]^+^ (calcd. for C_52_H_90_N_7_O_18_^+^, 1100.6337).

Streptodepsipeptide P11A (**2**): white amorphous powder; molecular formula C_53_H_88_N_6_O_18_; UV (MeOH, photodiode array), *λ*max 220 nm; ${\left[\alpha \right]}_{D}^{20}$ +34.0° (*c* 0.05, CHCl_3_) (lit. +21.6°, [6]); HR-ESI-MS *m/z* 1114.6486 [M + NH_4_]^+^ (calcd. for C_53_H_92_N_7_O_18_^+^, 1114.6493).

Valinomycin (**3**): white amorphous powder; molecular formula C_54_H_90_N_6_O_18_; UV (MeOH, photodiode array), *λ*max 210 nm; ${\left[\alpha \right]}_{D}^{20}$ +29.7° (*c* 0.60, CHCl_3_) (lit. +18.6°, [6]); HR-ESI-MS *m/z* 1128.6659 [M + NH_4_]^+^ (calcd. for C_54_H_94_N_7_O_18_^+^, 1128.6650).

Streptodepsipeptide SV21 (**4**): white amorphous powder; molecular formula C_55_H_92_N_6_O_18_; UV (MeOH, photodiode array), *λ*max 210 nm; ${\left[\alpha \right]}_{D}^{20}$ +15.0° (*c* 0.10, CHCl_3_); HR-ESI-MS *m/z* 1142.6804 [M + NH_4_]^+^ (calcd. for C_55_H_96_N_7_O_18_^+^, 1142.6806); for the ^13^C and ^1^H NMR data, see Table 2. 

### 3.2. Antimicrobial Assay

The panel of test microorganisms consisted of the following multi drug-resistant (MDR) bacteria: Gram-negative *Escherichia coli* (DSM 1116) and *Pseudomonas aeruginosa* (PA16); Gram-positive *Bacillus subtilis* (DSM 10), *Staphylococcus aureus* (DSM 346), and *Mycobacterium smegmatis* (ATCC 7000048); yeasts *Candida albicans* (DSM 1665) and *Rhodotorula glutinis* (DSM 10134); and filamentous fungi *Mucor hiemalis* (DSM 2656). The positive controls were nystatin, oxytetracyclin, kanamycin, and gentamycin, with a concentration of 1 mg/mL. Compounds **1**–**4** were prepared in a concentration of 1 mg/mL MeOH. The activity of the samples was observed by using the microdilution technique in 96 well plates for tissue cultures (TPP). As much as 20 µL of the MeOH solution of each compound (1 mg/mL) was mixed with 300 µL of bacterial or fungal suspension, except for the positive controls where 2 µL of either oxytetracycline, kanamycin, and gentamycin in 300 µL of bacterial suspension was used. The samples were tested in seven 1:2 serial dilution steps (concentrations A to H) in 96-well plates. Bacteria were cultivated in Mueller–Hinton bouillon (Roth) and fungi/yeasts in MYC medium (1.0% phytone peptone, 1.0% glucose, and 1.19% HEPES, pH 7.0). Start OD_600_ was 0.01 for *B. subtilis*, *E. coli,* and *S. aureus*; start OD_548_ was 0.1 for *M. hiemalis*, *C. albicans*, *R. glutinis*, *M. smegmatis,* and *P. aeruginosa*. The test organisms were cultivated at 30 °C and 160 rpm overnight.

### 3.3. Inhibitory Effects on Hepatitis C Virus (HCV) Infectivity

The anti-HCV assay was performed as mentioned in [5]. In brief, Huh7.5 cells stably expressing Firefly luciferase (Huh7.5 Fluc) were cultured in Dulbecco’s modified minimum essential medium (DMEM, Gibco, Thermo Fisher Scientific, Schwerte, Germany) and maintained in a 37 °C environment with 5% CO_2_ supply. Samples were added to the cells, and then the cells were infected with Jc1-derived *Renilla* reporter viruses. Compounds and the positive control (Epigallocatechin gallate, EGCG) were tested at a concentration of 10 µg/mL. *Renilla* and *Firefly luciferase* activities from the infected cells were measured on a Berthold Technologies Centro XS3 Microplate Luminometer (Bad Wildbad, Germany) as indicators of viral genome replication and cell viability, respectively.

## Figures and Tables

**Table 1:** MS^1^ data analysis results of the isolated compounds. The exact masses from the MS^1^ spectra of compounds **1**–**4** were compared against the MarinLit database (±0.01 Dalton).

Compound	Exact Mass, Observed MS^1^	Results in MarinLit	References
**1**	1100.6332 [M + NH_4_]^+^ 1082.5988 [M]	Streptodepsipeptide P11B ([M] = 1082.5999)	[6]
**2**	1114.6486 [M + NH_4_]^+^ 1096.6142 [M]	Streptodepsipeptide P11A ([M] = 1096.6155)	[6]
**3**	1128.6659 [M + NH_4_]^+^ 1110.6315 [M]	valinomycin ([M] = 1110.6311)	[6,9]
**4**	1142.6804 [M + NH_4_]^+^ 1124.6460 [M]	Not yet reported	

**Table 2:** ^1^H and ^13^C NMR data for compound **4**.

Position	NH		CO	Cα		Cβ		Cγ1		Cγ2		Cδ		Cβ-Me	
	δ_C_	δ_H_ (*J* in Hz)	δ_C_	δ_C_	δ_H_ (*J* in Hz)	δ_C_	δ_H_ (*J* in Hz)	δ_C_	δ_H_ (*J* in Hz)	δ_C_	δ_H_ (*J* in Hz)	δ_C_	δ_H_ (*J* in Hz)	δ_C_	δ_H_ (*J* in Hz)
*D*-Val (1)	118.4	7.88	169.9	58.5	4.19	28.4	2.34	19.0	1.05	19.1	0.96				
*D*-Val (2)	118.4	7.83	170.2	58.7	4.13	28.4	2.33	19.0	1.05	19.1	0.96				
*D*-Val (3)	118.0	7.80	170.1	58.7	4.14	28.4	2.32	19.0	1.05	19.1	0.96				
*L*-Lac (1)			172.4	70.2	5.30	17.0	1.45								
*L*-Lac (2)			172.4	70.2	5.30	17.0	1.45								
*L*-Lac (3)			172.4	70.2	5.30	17.0	1.45								
*L*-Val (1)	118.8	7.75	171.5	60.2	4.00	28.3	2.26	19.6	1.08	19.3	0.96				
*L*-Val (2)	118.8	7.69	171.8	60.3	3.97	28.3	2.23	19.6	1.07	19.3	0.95				
*L*-Val (3)	118.7	7.67	171.7	60.2	3.99	28.3	2.24	19.6	1.08	19.3	0.96				
*D*-Hiv (1)			170.5	78.5	5.02	30.2	2.35	16.5	0.98						
*D*-Hiv (2)			170.7	78.5	5.02	30.2	2.35	16.5	0.98						
Hmpa			170.9	76.7	5.17	36.7	2.11	26.1	1.41	26.1	1.29	11.7	0.93	14.0	0.96

**Table 3:** Minimum inhibitory concentration (MIC) of valinomycin (**3**) and analogues (**1**, **2**, and **4**) against different human pathogenic MDR bacteria and fungi. The activities of the tested compounds were compared to the positive controls oxytetracyclin, kanamycin, gentamycin, and nystatin.

Tested Strains:	MIC (µg mL^−1^)							
	Gram-Positive			Gram-Negative		Fungi		
	*Bs*	*Sa*	*Ms*	*Ec*	*Pa*	*Ca*	*Mh*	*Rg*
Compound:								
Streptodepsipeptide P11B (**1**)	-	-	-	-	-	-	16.6	33.3
Streptodepsipeptide P11A (**2**)	-	-	-	-	-	-	8.3	8.3
Valinomycin (**3**)	-	4.2	-	-	-	-	2.1	4.2
Streptodepsipeptide SV21 (**4**)	33.3	16.6	-	-	-	-	16.6	16.6
Positive controls:								
Oxytetracycline	8.3	0.2	n.t.	1.7	n.t.	n.t.	n.t.	n.t.
Kanamycin	n.t.	n.t.	3.3	n.t.	n.t.	n.t.	n.t.	n.t.
Gentamycin	n.t.	n.t.	n.t.	n.t.	0.4	n.t.	n.t.	n.t.
Nystatin	n.t.	n.t.	n.t.	n.t.	n.t.	33.3	16.6	4.2

Abbreviation for MDR bacterial strains and fungi: *Bs*: *Bacillus subtilis* (DSM 10); *Ca*: *Candida albicans* (DSM 1665); *Ec*: *Eschericia coli* (DSM 1116); *Mh*: *Mucor hiemalis* (DSM 2656); *Ms*: *Mycobacterium smegmatis* (ATCC 700084); *Pa*: *Pseudomonas aeruginosa* (PA14); *Sa*: *Staphylococcus aureus* (DSM 346); *Rg*: *Rhodoturula glutinis* (DSM 10134); -: not active; n.t.: not tested.

## Data Availability

The data presented in this study are available on request from the corresponding authors.

## Supplementary Materials

The following are available online at https://www.mdpi.com/1660-3397/19/2/81/s1. Figure S1. The HRMS spectra of compounds **1**–**4**; Figures S2–S5. Mirror-match of compounds **1**–**4** with valinomycin from the MASST GNPS database; Figures S6–S10. MS^2^ spectra of the compounds **1**–**4**; Tables S1–S4. ^1^H-NMR from [6]; Figures S11–S15. ^1^H-NMR of compounds **1**–**4** in CDCl_3_; Figure S16. 1D ^13^C-NMR spectrum of streptodepsipeptide SV21 (**4**) in CDCl_3_; Figure S17. 1D ^1^H-NMR spectrum of streptodepsipeptide SV21 (**4**) in CDCl_3_; Figure S18. Full HSQC spectrum of streptodepsipeptide SV21 (**4**).
