# Supplementary material for: Anti-Infective and Antiviral Activity of Valinomycin and Its Analogues from a Sea Cucumber-Associated Bacterium, Streptomyces sp. SV 21
Source: Mar Drugs. 2021 Feb 2;19(2):81. doi: 10.3390/md19020081 (PMC7912928; doi:10.3390/md19020081)
Supplement: Supplementary file 1 [file marinedrugs-19-00081-s001.pdf]

SUPPLEMENTARY MATERIAL

Figure S1. The HRMS spectra of compounds **1 – 4**.

Figure S2-S5. Mirror-match of compounds **1 – 4** with valinomycin from the MASST GNPS database.

Figure S6-S10. MS^2 spectra of the compounds **1 – 4.**

Table S1-S4. ^{1}H-NMR from the reference Ye et al. Phytochemistry 135 (2017), 151-159.

Figure S11-S15. ^{1}H-NMR of compounds **1 – 4** in $CDCl_3$.

Figure S16. Full ^{13}C-NMR spectrum of streptodepsipeptide SV21 (**4**) in $CDCl_3$.

Figure S17. Full ^{1}H-NMR spectrum of streptodepsipeptide SV21 (**4**) in $CDCl_3$.

Figure S18. Full HSQC spectrum of streptodepsipeptide SV21 (**4**).

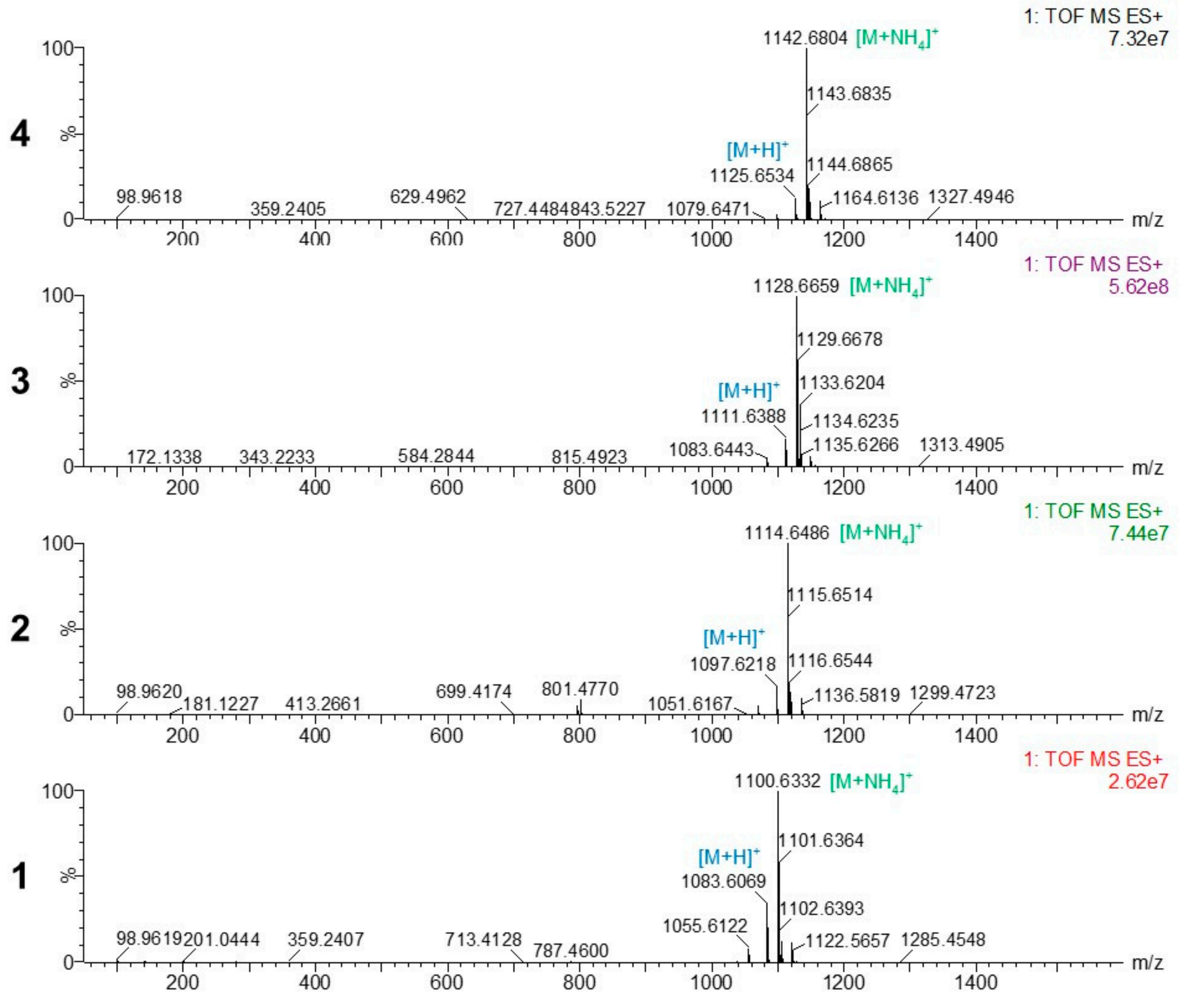

Figure S1. The HRMS spectra of compounds **1 - 4**.

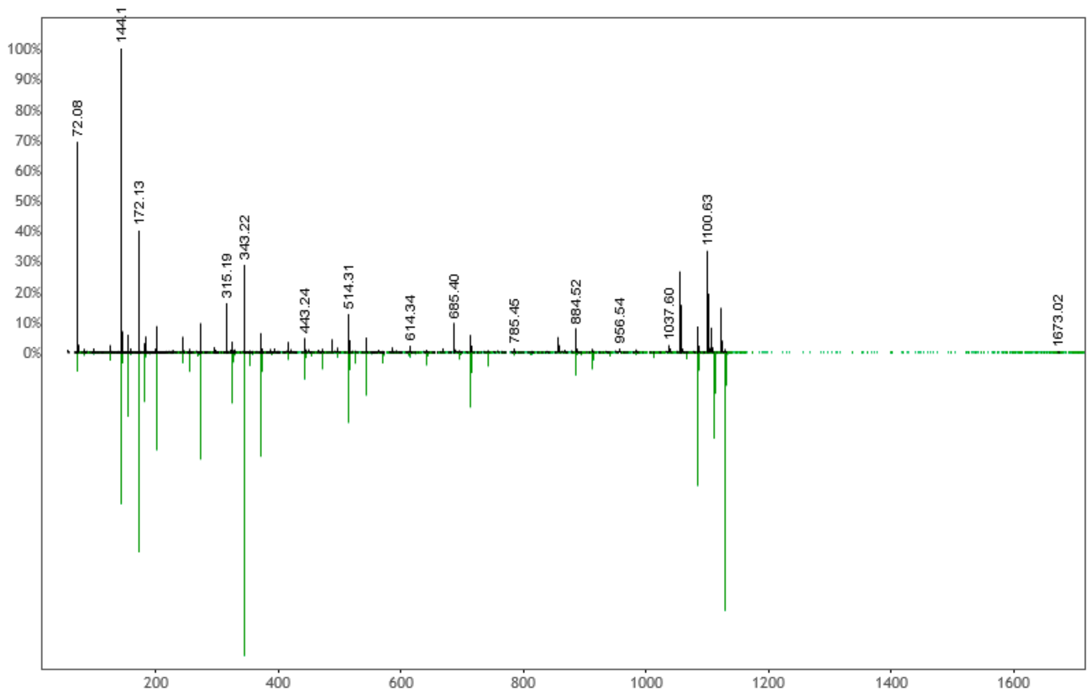

Figure S2. Mirror-match of compound **1** with valinomycin from the MASST GNPS database (library class: gold; cosine-score: 0.76; shared peaks: 44; mass-diff: 28.03; specMZ: 1100.63; libMZ: 1128.66).

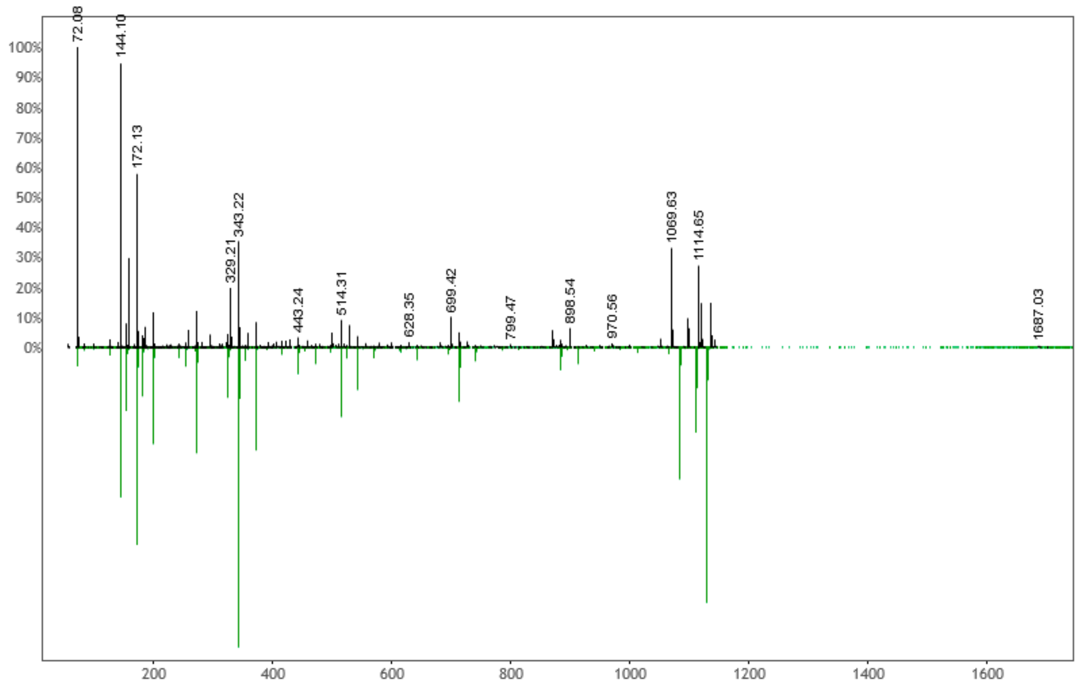

Figure S3. Mirror-match of compound **2** with valinomycin from the database (library class: gold; cosine-score: 0.77; shared peaks: 42; mass-diff: 14.01; specMZ: 1114.54; libMZ: 1128.66).

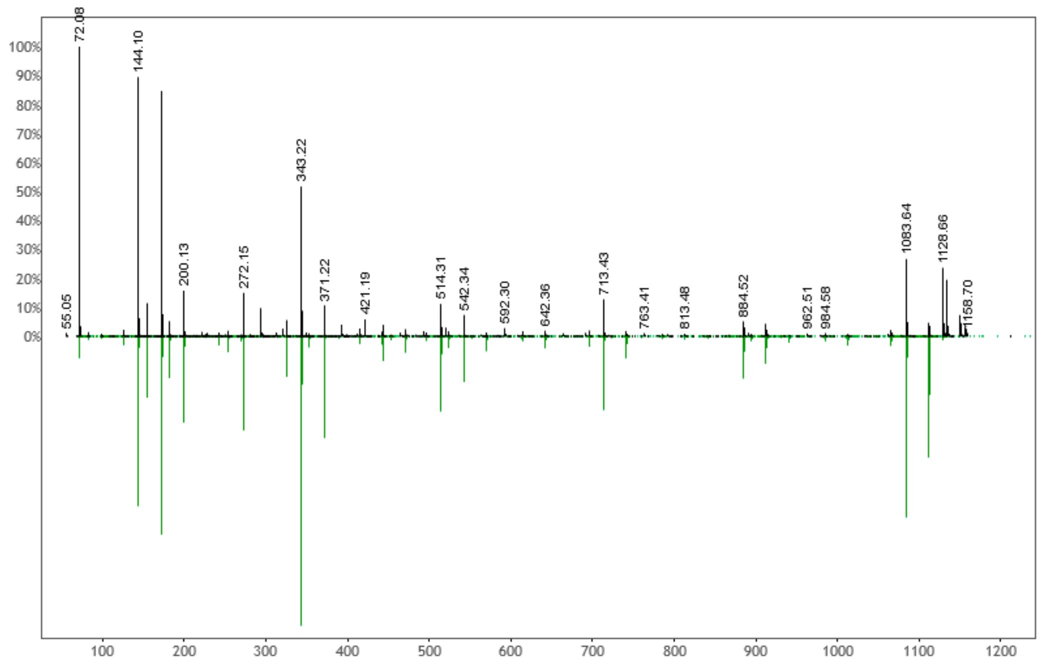

Figure S4. Mirror-match of valinomycin **(3)** with valinomycin from the database (library class: gold; cosine-score: 0.83; shared peaks: 49; mass-diff: 0.01; specMZ: 1128.67; libMZ: 1128.66).

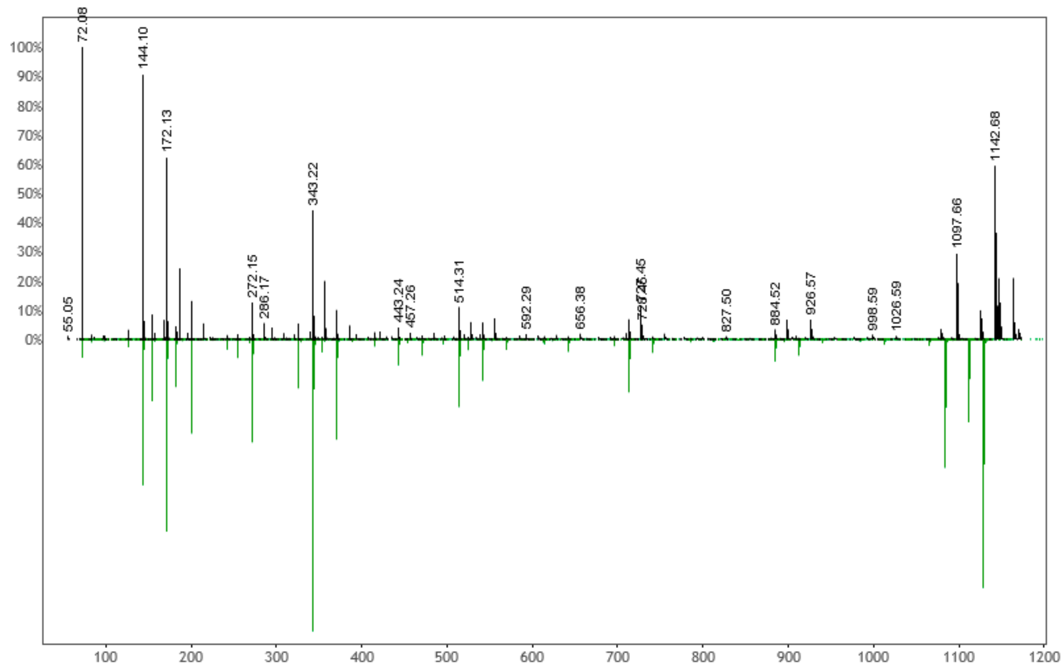

Figure S5. Mirror-match of compound **4** with valinomycin from the database (library class: gold; cosine-score: 0.77; shared peaks: 42; mass-diff: 14.02; specMZ: 1142.68; libMZ: 1128.66).

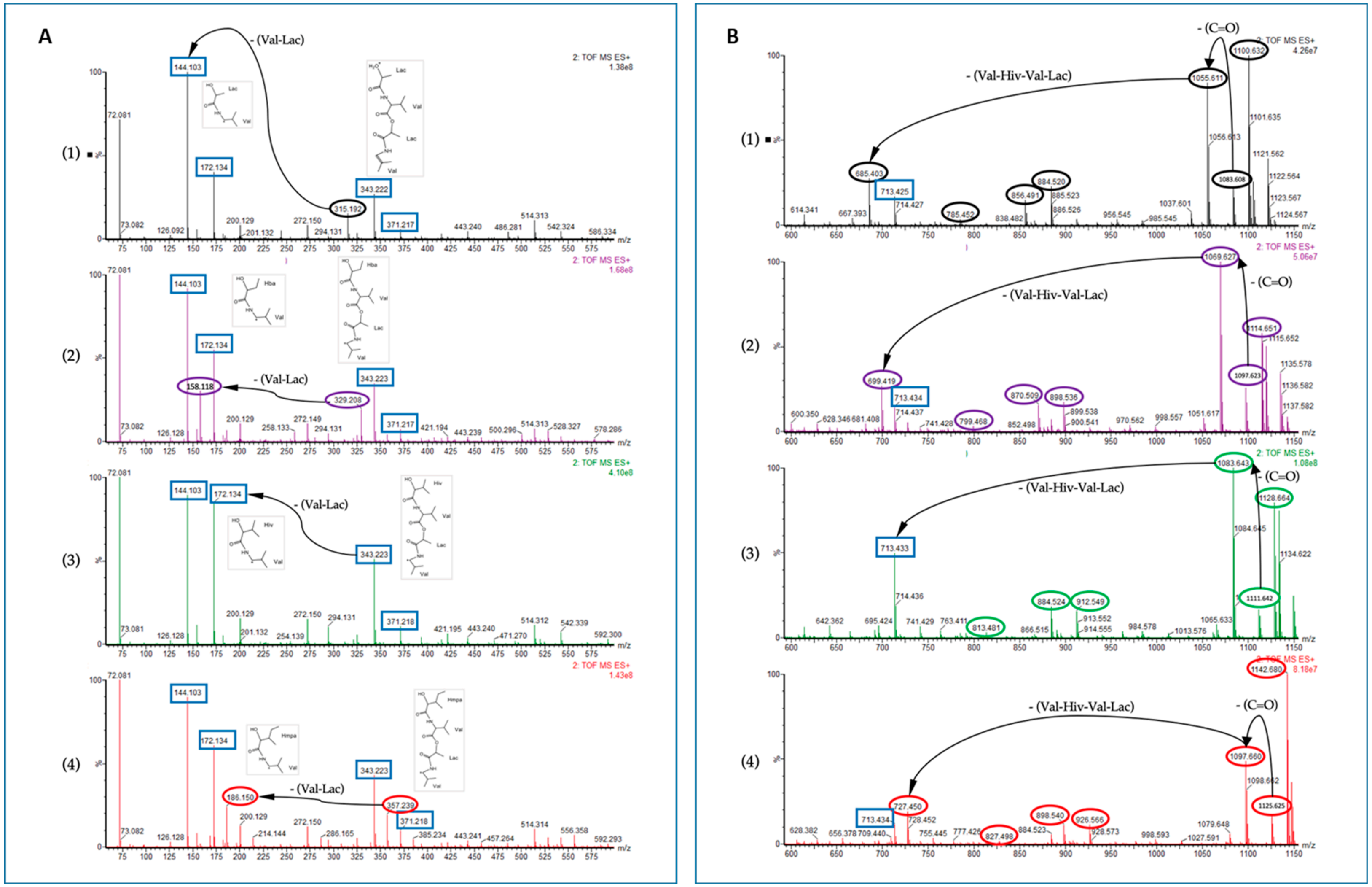

Figure S6. MS^2 spectra of the compounds **1 – 4**: (A)The mass window magnifies on the range between *m/z* 50 – *m/z* 600; and (B) on the range between *m/z* 600 – *m/z* 1150. Identical masses that occurred in all four spectra of the derivates of valinomycin were marked with a blue box, while the compound specific masses were highlighted in circles.

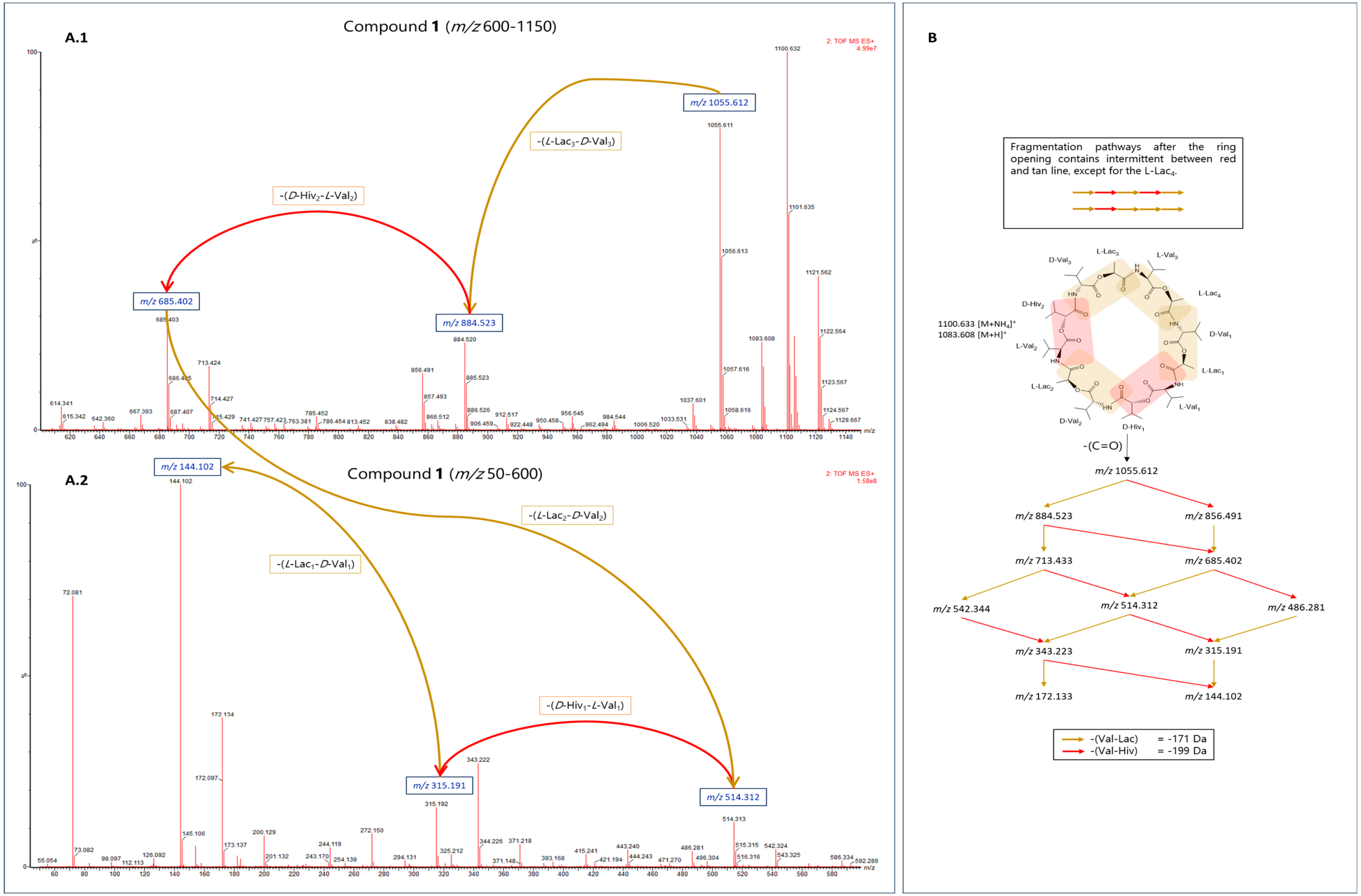

Figure S7. MS^2 spectra of compound **1**. (A.1) from *m/z* 600 – *m/z* 1150, (A.2) *m/z* 50 – *m/z* 600, and (B) the possible fragmentation pathways.

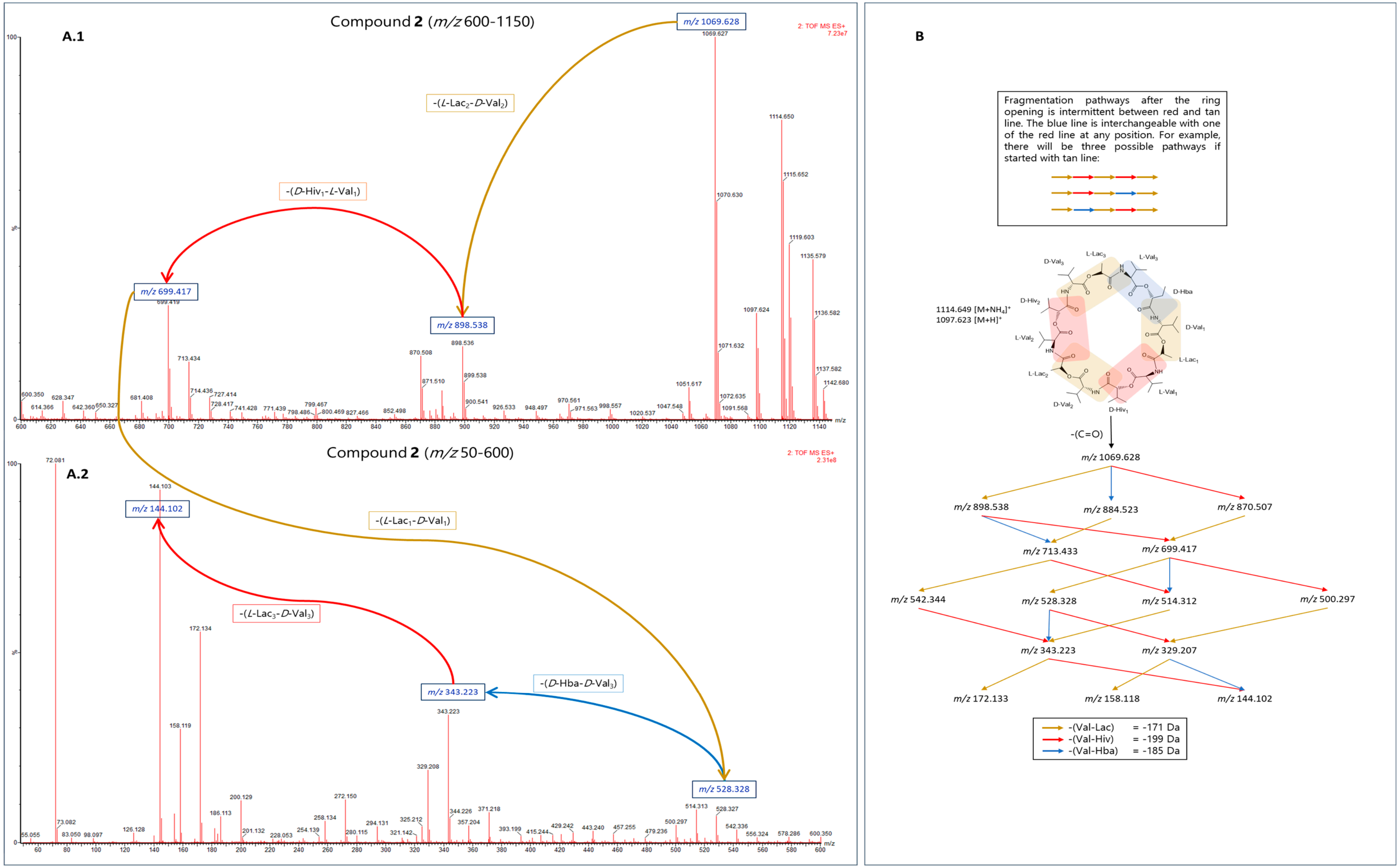

Figure S8. MS[2] spectra of compound **2**. (A.1) from *m/z* 600 – *m/z* 1150, (A.2) *m/z* 50 – *m/z* 600, and (B) the possible fragmentation pathways.

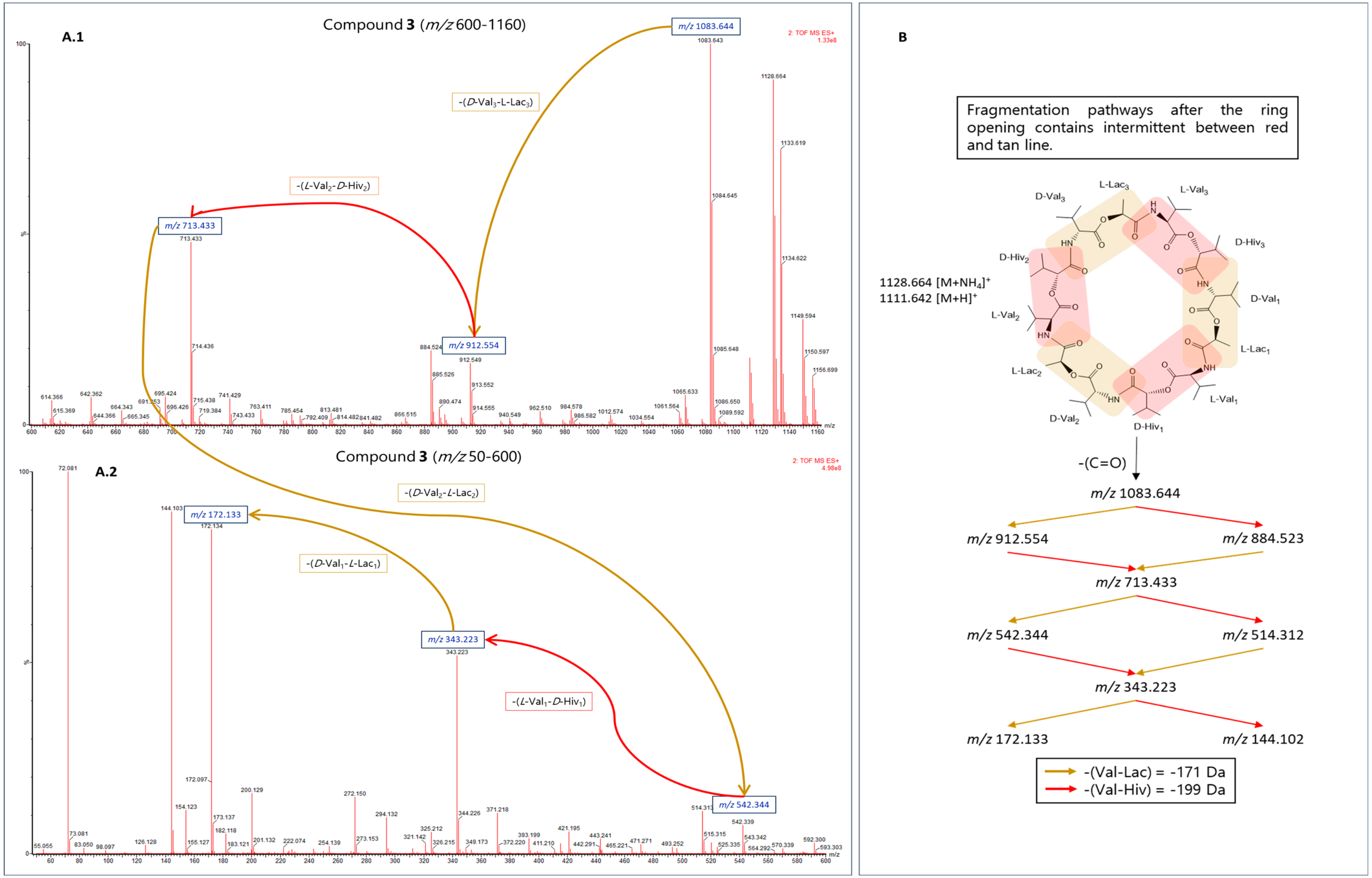

Figure S9. MS^2 spectra of valinomycin (**3**). (A.1) from *m/z* 600 – *m/z* 1150, (A.2) *m/z* 50 – *m/z* 600, and (B) the possible fragmentation pathways.

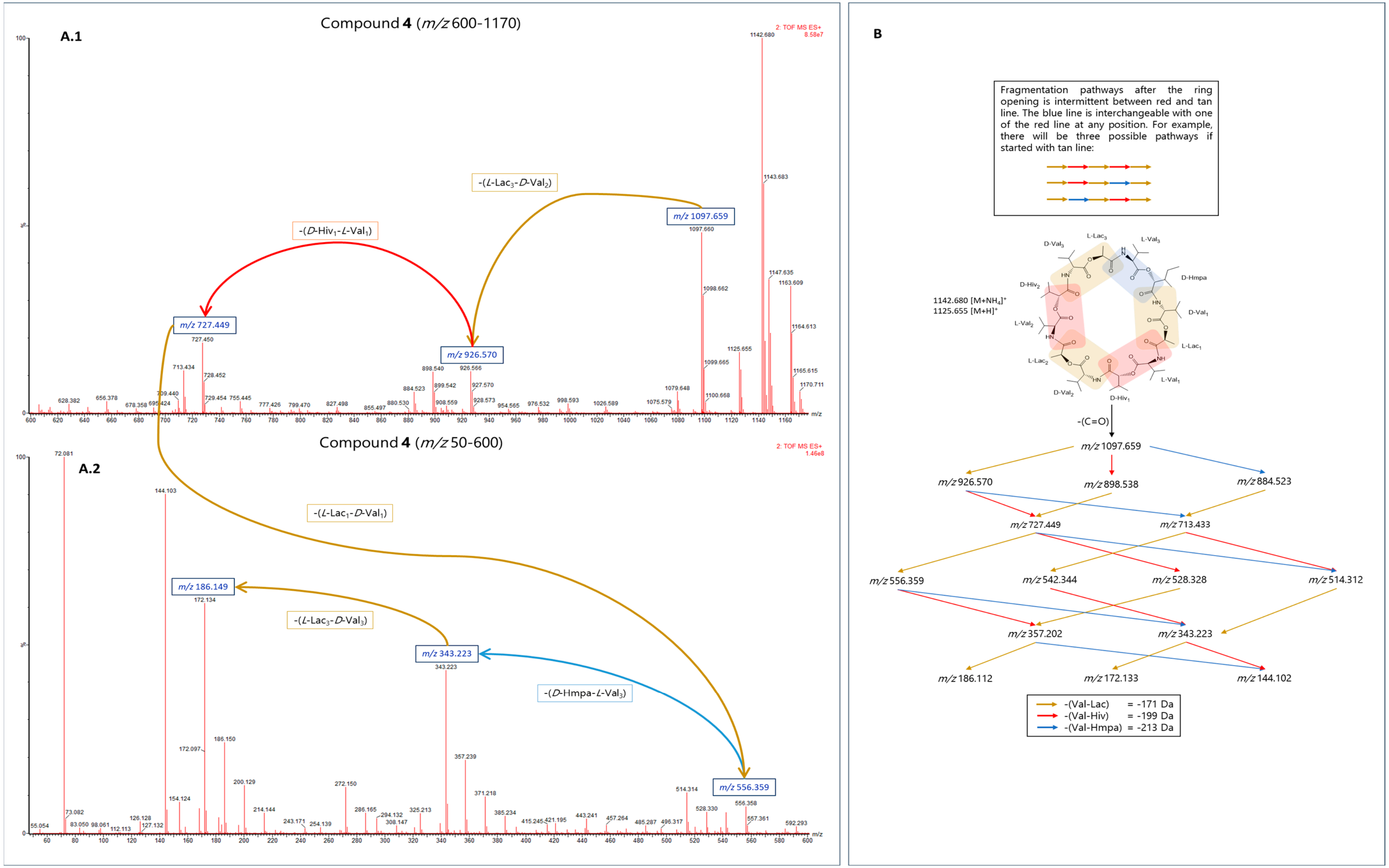

Figure S10. MS^2 spectra of compound **4**. (A.1) from *m/z* 600 – *m/z* 1150, (A.2) *m/z* 50 – *m/z* 600, and (B) the possible fragmentation pathways.

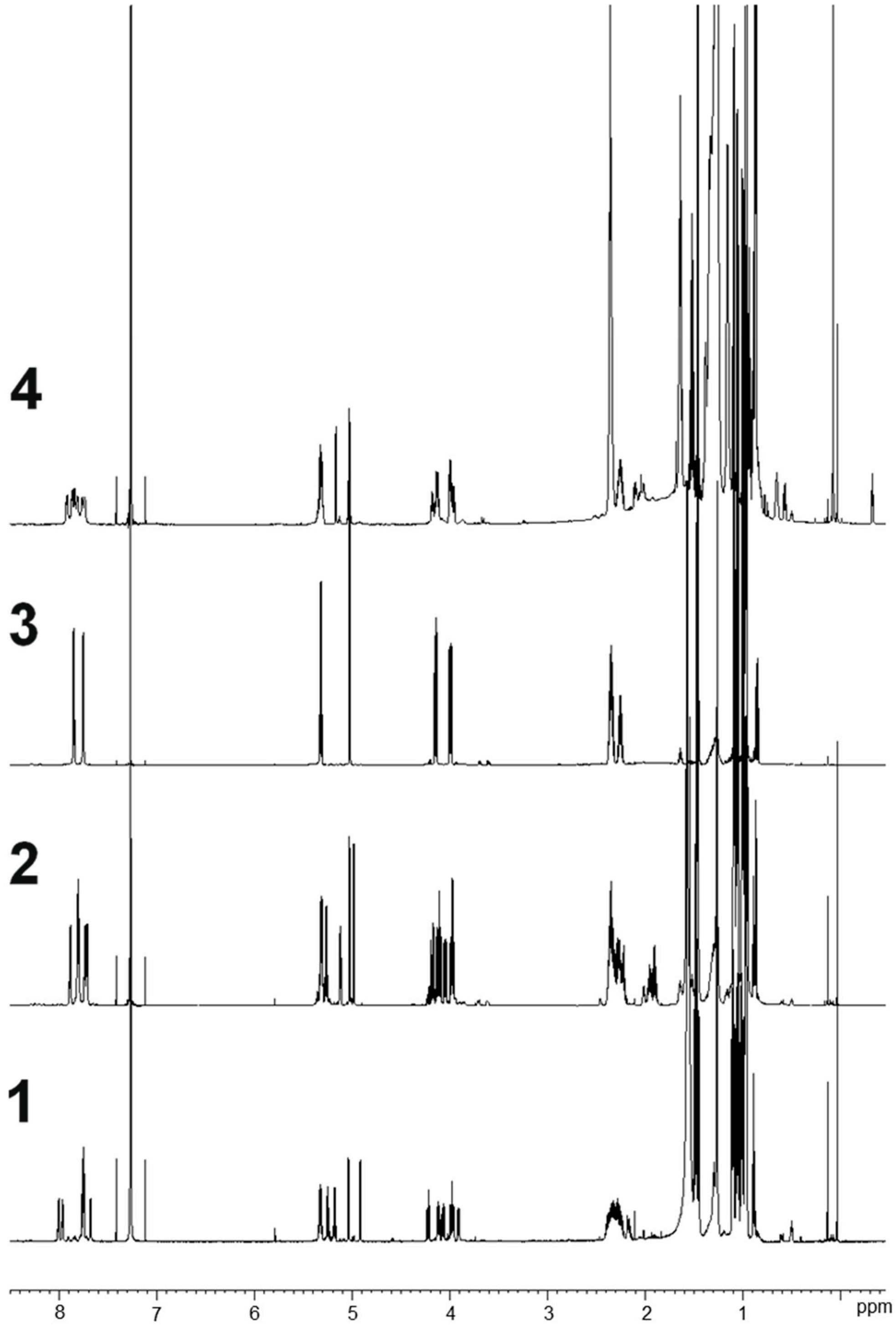

Figure S11. Full ^{1}H-NMR spectra of compounds **1** to **4.**

Table S1. ^{1}H-NMR at amide regions from the reference Ye et al. Phytochemistry 135 (2017), 151-159.

	Streptodepsipeptide P11B		Streptodepsipeptide P11A		Valinomycin	
		**δ_H, (*J* in Hz)**		**δ_H, (*J* in Hz)**		**δ_H, (*J* in Hz)**
NH	L-Val	7.74, d (5.9)	L-Val1	7.76, d (5.8)	L-Val	7.80, d (6.2)
		7.78, d (6.3)	L-Val2	7.84, d (6.0)	D-Val	7.88, d (8.1)
		7.96, d (8.2)	L-Val3	7.77, d (6.2)		
	D-Val	7.79, d (8.2)	D-Val1	7.83, d (8.0)		
		7.79, d (8.2)	D-Val2	7.83, d (8.0)		
		8.00, d (7.6)	D-Val3	7.89,d (7.9)		

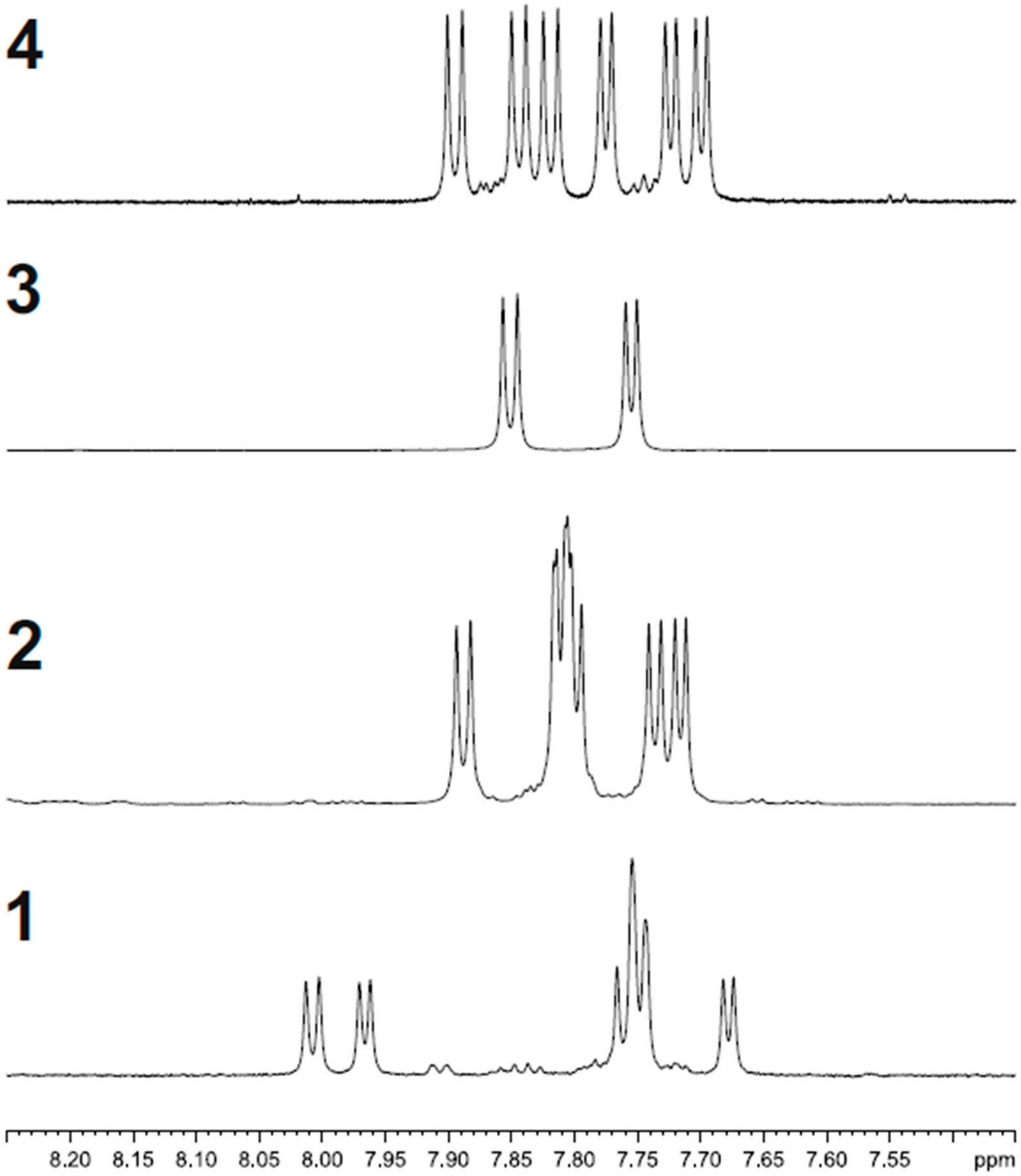

Figure S12. ^{1}H-NMR spectra at amide regions of compounds **1-4** in $CDCl_3$.

Table S2. ^{1}H-NMR at Hα regions from the reference Ye et al. Phytochemistry 135 (2017), 151-159.

	Streptodepsipeptide P11B		Streptodepsipeptide P11A		Valinomycin	
		**δ_H, (*J* in Hz)**		**δ_H, (*J* in Hz)**		**δ_H, (*J* in Hz)**
α-CH-O	D-Hiv	4.94, d (3.2)	D-Hiv_1	4.99, d (3.2)	D-Hiv	5.02, d (3.1)
		5.04, d (3.0)	D-Hiv_2	5.02, d (3.2)		
	L-Lac	5.22, q (7.0)	D-Hba	5.11, dd (6.6, 4.3)		
		5.20, q (7.0)	L-Lac_1	5.33, q (6.8)	L-Lac	5.32, q (7.0)
		5.32, q (7.0)	L-Lac_2	5.27, q (6.8)		
		5.31, q (7.0)	L-Lac_3	5.33, q (6.8)		
α-CH-N	L-Val	3.91, dd (10.0, 5.9)	L-Val_1	3.96, dd (10.0, 5.8)	L-Val	3.96, dd (10.2, 6.2)
		3.96, dd (10.0, 6.3)	L-Val_2	3.97, dd (10.0, 6.0)		
		4.01, dd (9.8, 6.0)	L-Val_3	4.01, dd (10.0, 6.2)		
	D-Val	4.05, dd (10.0, 7.6)	D-Val_1	4.13, dd (9.2, 8.0)	D-Val	4.10, dd (10.0, 8.1)
		4.07, dd (10.0, 8.2)	D-Val_2	4.08, dd(9.2, 8.0)		
		4.16, dd (9.8, 8.2)	D-Val_3	4.08, dd(9.2, 7.9)		

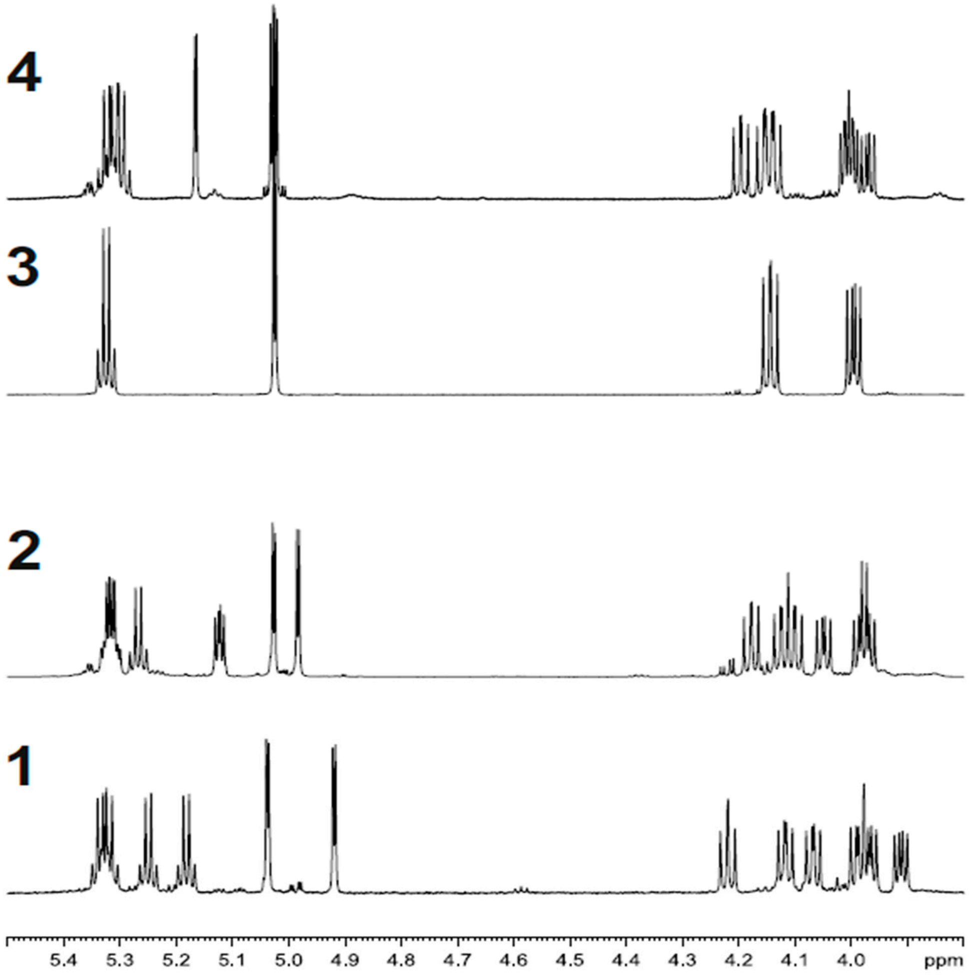

Figure S13. ^{1}H-NMR spectra at Hα regions of compounds **1-4** in $CDCl_3$.

Table S3. ^{1}H-NMR at Hβ regions from the reference Ye et al. Phytochemistry 135 (2017), 151-159.

	Streptodepsipeptide P11B		Streptodepsipeptide P11A		Valinomycin	
		**δ_H, (*J* in Hz)**		**δ_H, (*J* in Hz)**		**δ_H, (*J* in Hz)**
β-CH_2			D-Hba	1.91, m		
β-CH	D-Hiv	2.36, m	D-Hiv	2.37, m	D-Hiv	2.33, m
	Val	2.17-2.40	Val	2.27-2.35	L-Val	2.23, m
					D-Val	2.36, m

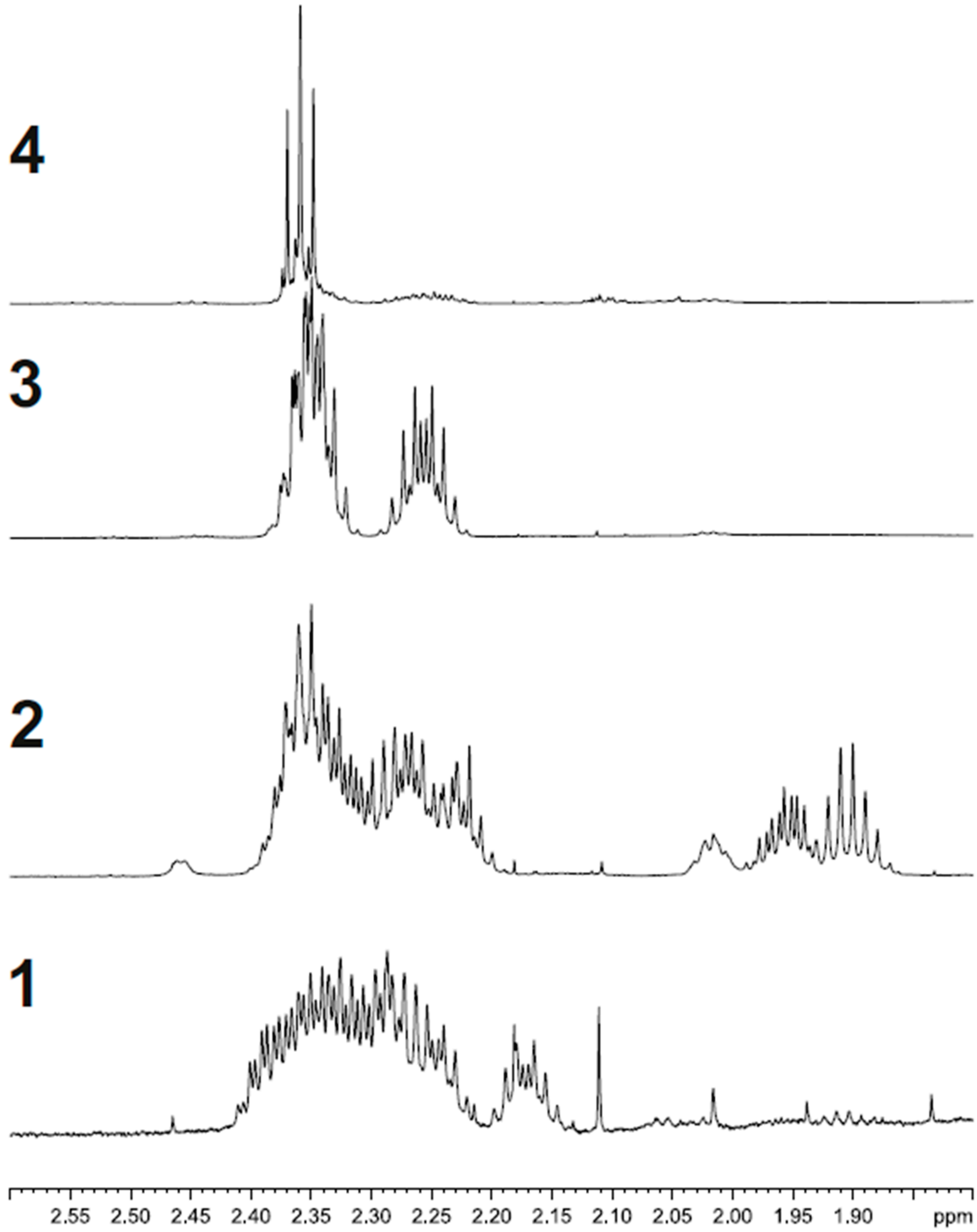

Figure S14. ^{1}H-NMR spectra at Hβ regions of compounds **1-4** in $CDCl_3$.

Table S4. ^{1}H-NMR at methyl regions from the reference Ye et al. Phytochemistry 135 (2017), 151-159.

	Streptodepsipeptide P11B		Streptodepsipeptide P11A		Valinomycin	
		**δ_H, (*J* in Hz)**		**δ_H, (*J* in Hz)**		**δ_H, (*J* in Hz)**
β-CH_3	L-Lac	1.44-1.49	L-Lac	1.44-1.47	L-Lac	1.44, d (7.0)
γ-CH_3	Val, D-Hiv	0.95-1.10	Val, D-Hiv	0.95-1.10	L-Val	1.08, d (6.6)
						0.95, d (6.6)
					D-Val	0.95, d (6.6)
						1.04, d 6.6)
	D-Hiv	0.98	D-Hiv	0.98	D-Hiv	0.98, d (6.6)
			D-Hba	0.95		0.96, d (6.6)

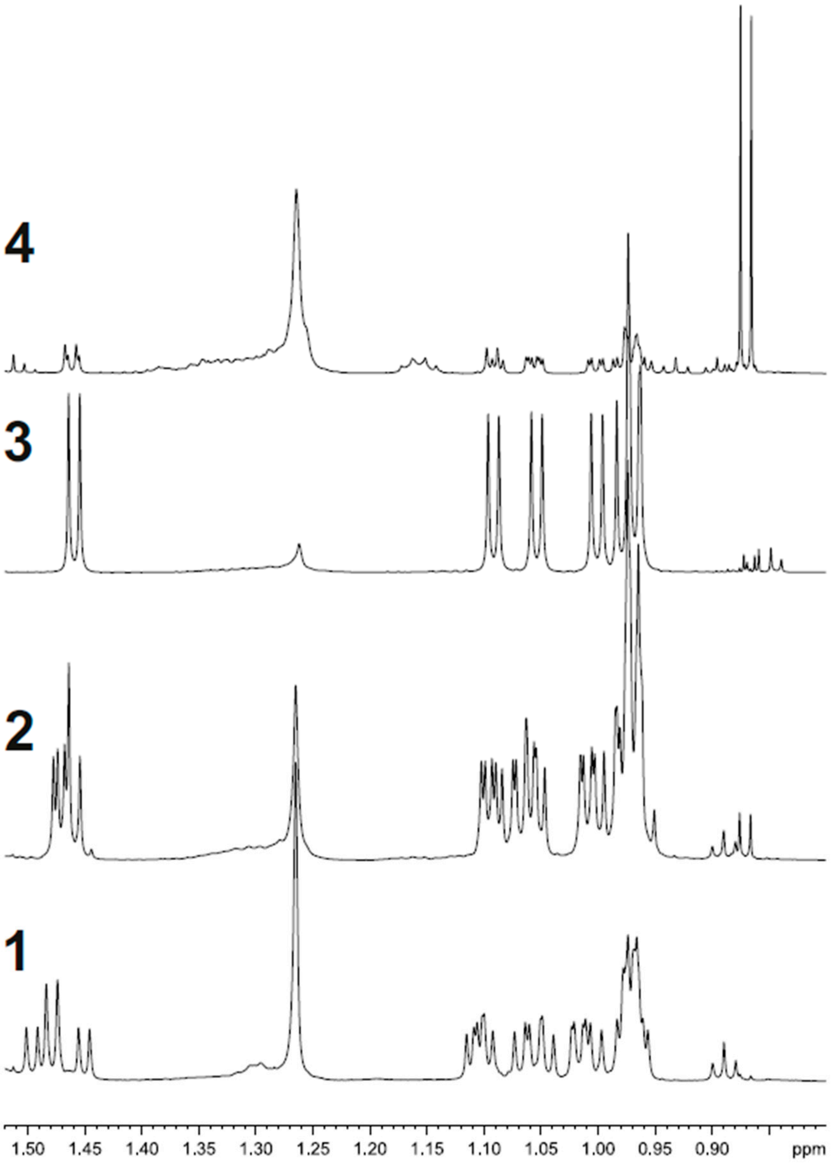

Figure S15. ^{1}H-NMR spectra at methyl regions of compounds **1-4** in $CDCl_3$.

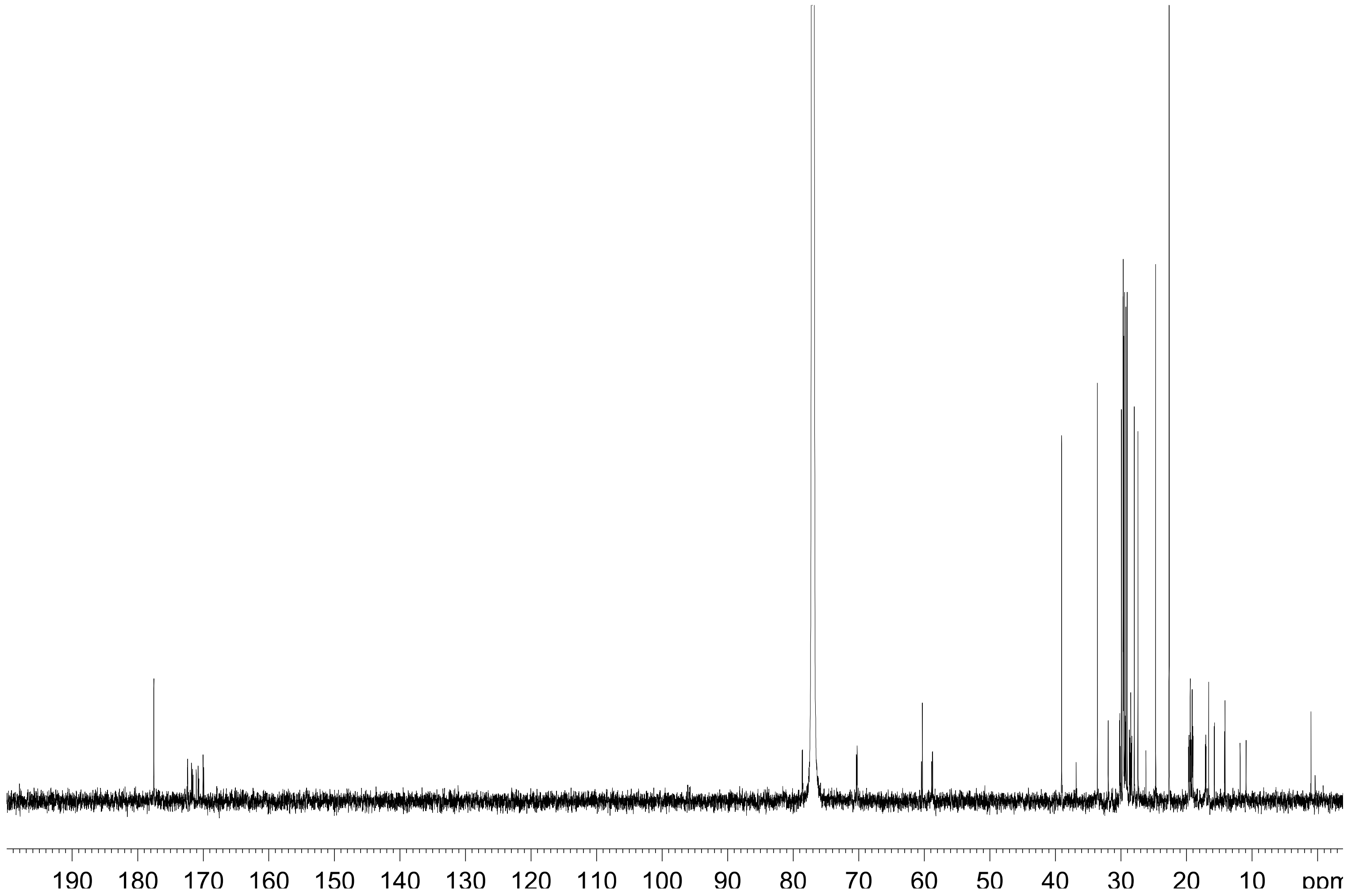

Figure S16. Full ^{13}C-NMR spectrum of streptodepsipeptide SV21 (**4**) in $CDCl_3$.

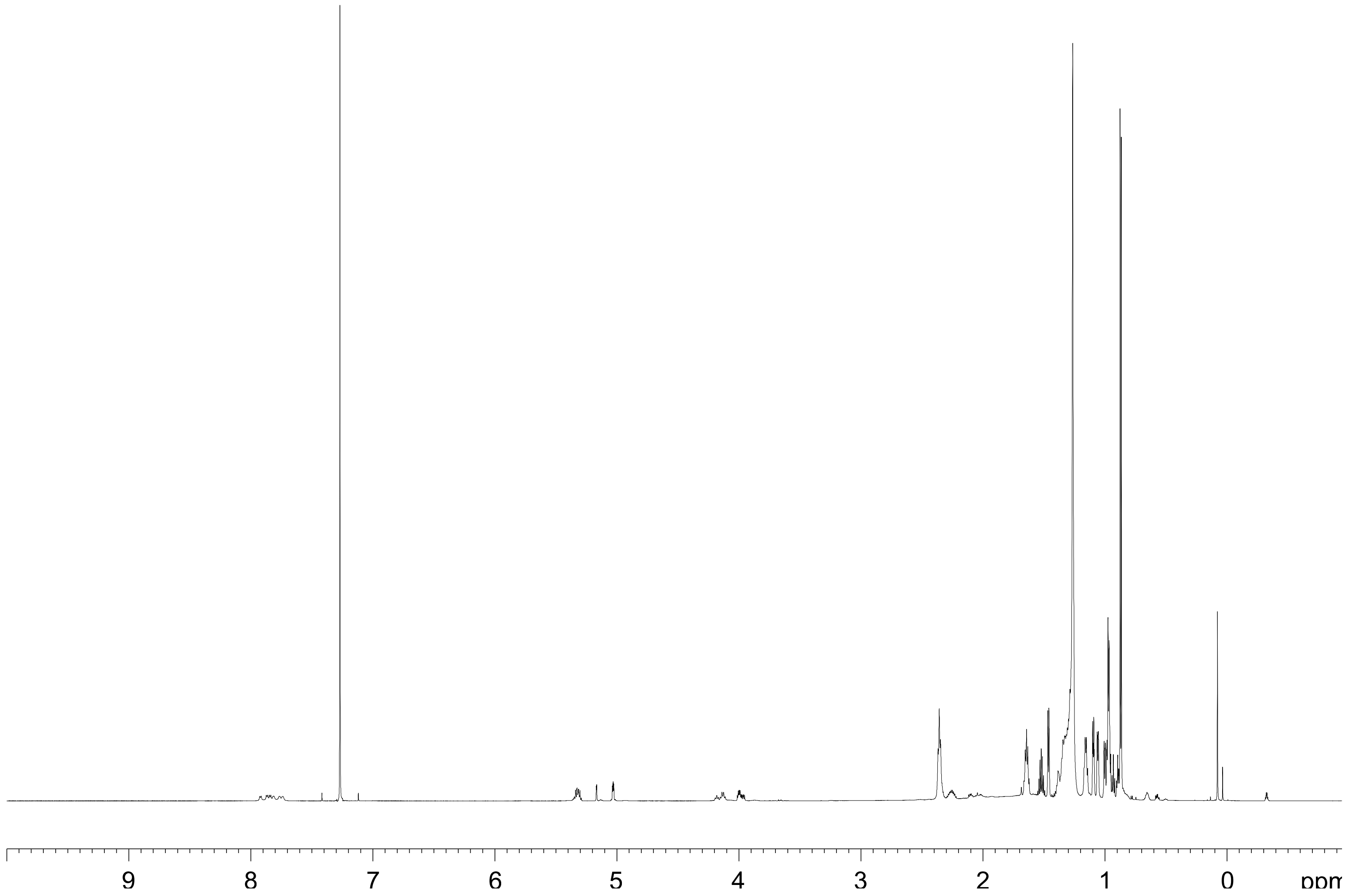

Figure S17. Full ^{1}H-NMR spectrum of streptodepsipeptide SV21 (**4**) in $CDCl_3$.

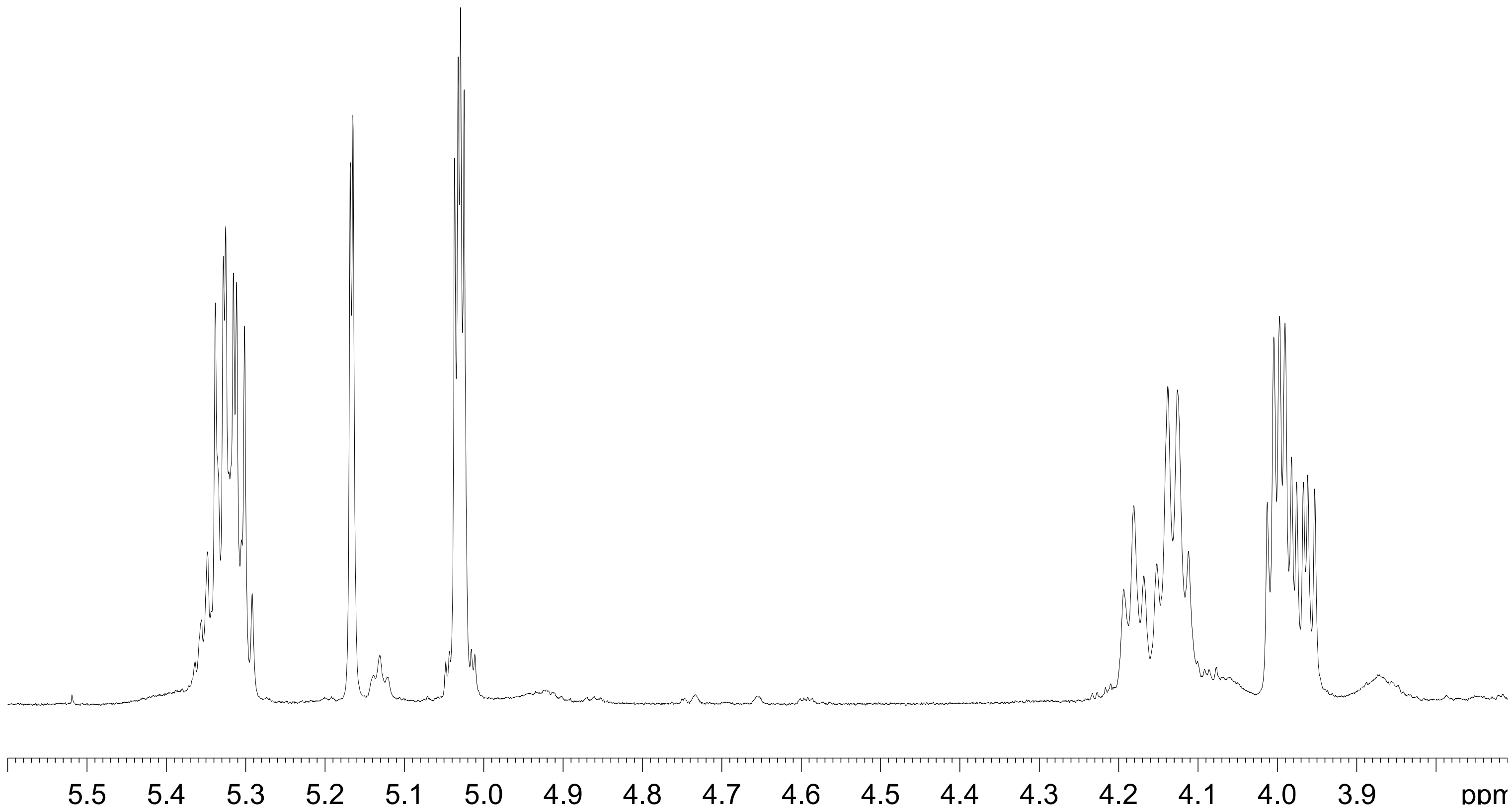

Figure S17.1: Hα region of the 1D ^{1}H-NMR spectrum of streptodepsipeptide SV21 (**4**) in $CDCl_3$.

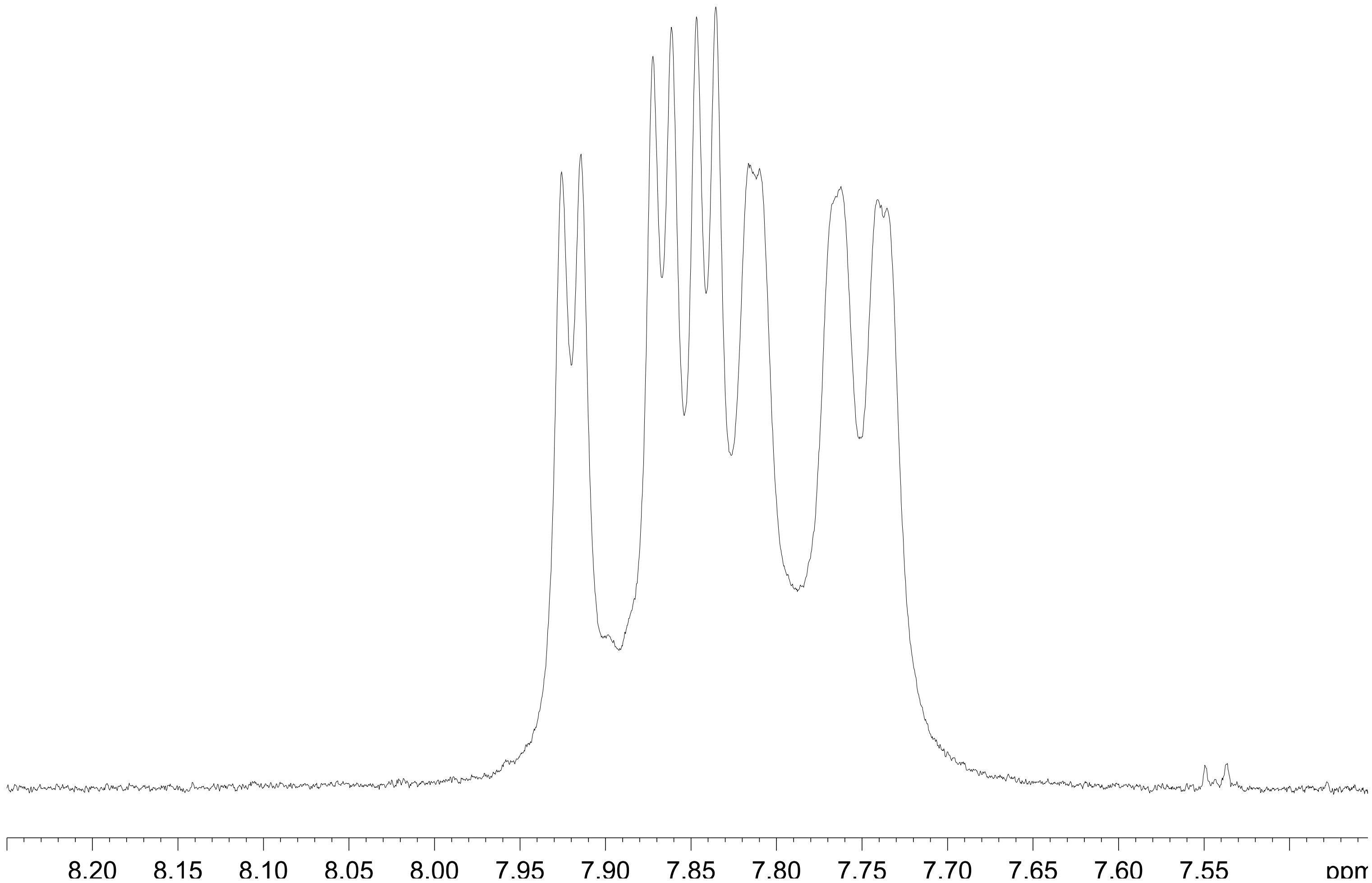

Figure S17.2. Amide regions of the 1D ^{1}H-NMR spectrum of streptodepsipeptide SV21 (**4**) in $CDCl_3$.

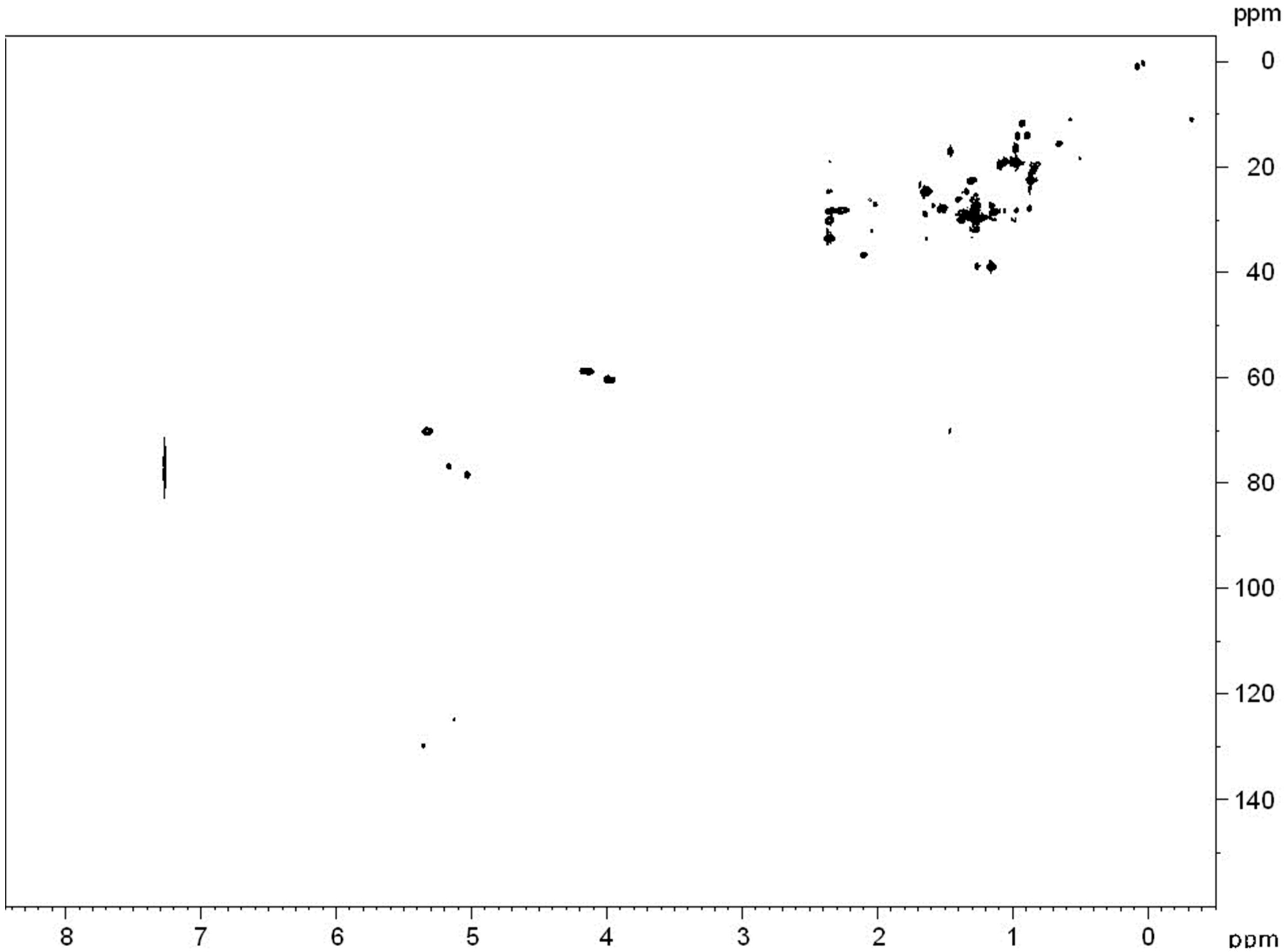

Figure S18. Full HSQC spectrum of streptodepsipeptide SV21 (**4**).
